# Cerebrospinal fluid proteome evaluation in major depressive disorder by mass spectrometry

**DOI:** 10.1186/s12888-020-02874-9

**Published:** 2020-10-01

**Authors:** Avery D. Franzen, Tukiet T. Lam, Kenneth R. Williams, Angus C. Nairn, Ronald S. Duman, Monica Sathyanesan, Vikas Kumar, Linda L. Carpenter, Samuel S. Newton

**Affiliations:** 1 Basic Biomedical Science Department, University of South Dakota, 414 E Clark St, Vermillion, SD 57069 USA; 2 Yale/Keck MS & Proteomics Resource, 300 George Street, Ste G001, New Haven, CT 06511 USA; 3 Yale/NIDA Neuroproteomics Center, 300 George Street, New Haven, CT 06511 USA; 4 Molecular Biophysics and Biochemistry, Yale University School of Medicine, New Haven, CT 067511 USA; 5 Yale Psychiatry, 34 Park Street, New Haven, CT 06508 USA; 6 Sioux Falls V A Health Care System, Sioux Falls, SD 57105 USA; 7 Mass Spectrometry & Proteomics Core Facility, University of Nebraska Medical Center, 985875 Nebraska Medical Center, Omaha, NE 68198 USA; 8 Brown Department of Psychiatry and Human Behavior, Butler Hospital, 345 Blackstone Boulevard, Providence, RI 02906 USA

**Keywords:** Cerebrospinal fluid, Proteomics, Label free quantitation, Major depressive disorder, Depression, Inflammation, Metabolism, Interleukin 6, Oncostatin M, STAT3

## Abstract

**Background:**

Depression affects approximately 7.1% of the United States population every year and has an annual economic burden of over $210 billion dollars. Several recent studies have sought to investigate the pathophysiology of depression utilizing focused cerebrospinal fluid (CSF) and serum analysis. Inflammation and metabolic dysfunction have emerged as potential etiological factors from these studies. A dysregulation in the levels of inflammatory proteins such as IL-12, TNF, IL-6 and IFN-γ have been found to be significantly correlated with depression.

**Methods:**

CSF samples were obtained from 15 patients, seven with major depressive disorder and eight age- and gender-matched non-psychiatric controls. CSF protein profiles were obtained using quantitative mass spectrometry. The data were analyzed by Progenesis QI proteomics software to identify significantly dysregulated proteins. The results were subjected to bioinformatics analysis using the Ingenuity Pathway Analysis suite to obtain unbiased mechanistic insight into biologically relevant interactions and pathways.

**Results:**

Several dysregulated proteins were identified. Bioinformatics analysis indicated that the potential disorder/disease pathways include inflammatory response, metabolic disease and organismal injury. Molecular and cellular functions that were affected include cellular compromise, cell-to-cell signaling & interaction, cellular movement, protein synthesis, and cellular development. The major canonical pathway that was upregulated was acute phase response signaling. Endogenous upstream regulators that may influence dysregulation of proinflammatory molecules associated with depression are interleukin-6 (IL-6), signal transducer and activator of transcription 3 (STAT3), oncostatin M, PR domain zinc finger protein 1 (PRDM1), and peroxisome proliferator-activated receptor gamma coactivator 1-alpha (PPARGC1A).

**Conclusions:**

The proteome profiling data in this report identifies several potential biological functions that may be involved in the pathophysiology of major depressive disorder. Future research into how the differential expression of these proteins is involved in the etiology and severity of depression will be important.

## Background

According to the National Institute of Mental Health (NIMH), 17.3 million U.S. adults had at least one episode of Major Depressive Disorder (MDD) in 2017 [1]. This number represents 7.1% of the United States population and that number is only expected to rise. The total economic burden of MDD in the U.S. was estimated to be $210.5 billion dollars in 2010 [2]. MDD is a challenging disease to study as it is a multifaceted and polygenic disorder with environmental influences. Several methods have been employed on plasma, saliva, and CSF to understand the deeper mechanisms of MDD including transcriptomics [3], DNA sequencing [4], and genome wide association studies (GWAS) [5]. Recent research has pointed to possible correlations of depression with processes such as inflammation [6, 7] and metabolic disease [8]. Inflammatory proteins such as IL-12, TNF, IL-6, IFN-γ, IL-9, IL-17A, and IL-10 have been reported as being elevated in MDD patients [9] and studies regarding the role of metabolism in the disease are underway. Several groups have used the above approaches to investigate biomarkers circulating in the peripheral blood [10, 11] or saliva. However, cerebrospinal fluid (CSF) may be a more promising biomarker source because of its proximity to and direct interactions with brain tissue [12]. Similar to plasma studies, it shares the advantage over post-mortem brain tissue studies in that samples can be collected from live patients. Traditionally, CSF proteomics studies have employed 2D-gel electrophoresis, which is quantitative but requires relatively large quantities of protein that can only be identified post-analysis [13]. Mass spectrometry methods are ideal for CSF studies because their high sensitivity requires relatively low protein concentrations [14]. In this study, CSF samples from MDD patients and matching non-psychiatric patients were analyzed by quantitative mass spectrometry. The resulting data was subjected to bioinformatic analyses with Ingenuity Pathway Analysis to determine potential pathways involved in the pathophysiology of MDD.

## Methods

### Cerebrospinal fluid collection

Ten adult outpatients fulfilling DSM-IV criteria for unipolar MDD and ten non-psychiatric controls, selected to age and gender match the patient group, provided voluntary written informed consent to participate in this study. A demographic table for these patients has been included in Supplementary Table 1. The protocol was approved by the institutional review boards of Yale University (New Haven, CT) and Butler Hospital (Providence, RI), and conducted at both institutions. For a detailed description of subjects and CSF collection see [15]. Briefly, depressed patients with baseline Hamilton Depression Rating Scale score greater than 17 were recruited. Diagnostic interviews were used to determine the presence of unipolar MDD (patient group) or the absence of any current and lifetime DSM-IV Axis I disorder (controls). Individuals with any other major Axis I comorbidity were excluded. All participating subjects were medication-free for at least 2 weeks. MDD subjects underwent CSF sampling within 2 weeks prior to starting their clinical trial antidepressant treatment.

Efforts were taken to reduce anxiety and HPA axis arousal associated with the lumbar puncture (LP) procedure. Subjects were in a comfortable leaning-forward seated position on a bed and repeatedly encouraged to provide feedback in order to achieve a relatively pain-free LP by adjusting positioning and liberal application of local anesthetic. Procedure was terminated if CSF sample was not obtained by 30 min after the start of preparations. Collection of samples was completed when 10 patients in each group had successful lumbar punctures.

**Fig. 1:**
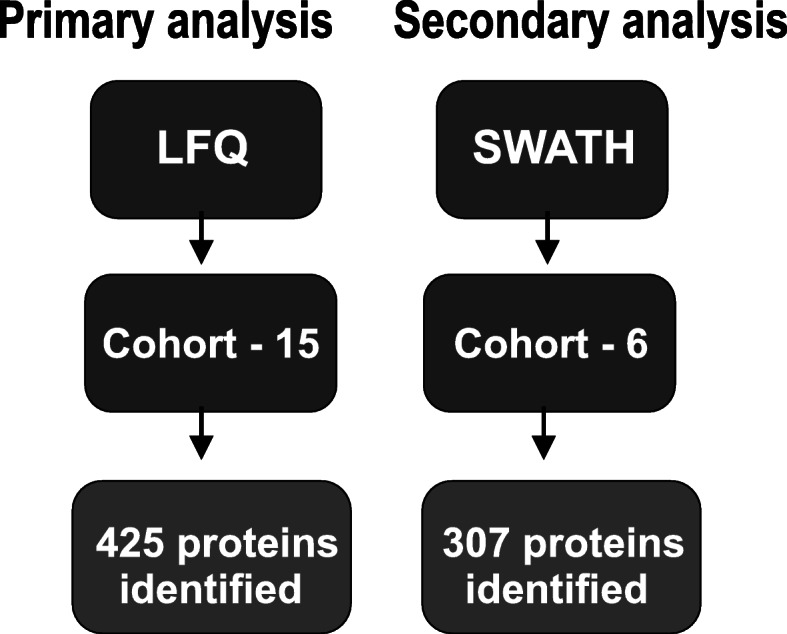
Workflow of mass spectrometry experiments. LTQ – Linear trap quadropole, SWATH – Sequential window acqusition of all theoretical fragment ion spectra

A total of 12 ml of clear CSF was collected and frozen at − 80 °C in 0.5 ml aliquots. In addition to the samples being clear and devoid of coloration, mass spectrometry revealed a negligible amount of hemoglobin alpha and beta changes with no significant difference between the two groups. Other blood specific and highly abundant blood proteins including catalase, peroxiredoxin, and carbonic anhydrase I were not detected in the CSF samples [16]. This gives us a high degree of confidence that blood contamination did not occur or is below detection sensitivity. A workflow of the MS experiments is shown in Fig. 1.

### Mass spectrometry detection and quantification of proteins

#### LabelFree analysis

5 μL of digested samples (EN or FT, at equal conc. ~ 0.1 μg/μL) are injected onto a nanoACQUITY™ UPLC™ in-line with an LTQ Orbitrap Elite MS system equipped with a Waters nanoACQUITY™ UPLC™ system, and uses a Waters Symmetry® C18 180 μm × 20 mm trap column and a 1.7 μm, 75 μm × 250 mm nanoAcquity™ UPLC™ column (35 °C) for peptide separation. The LC gradient and sequence of MS events are described below. Buffer A: 0.1% Formic Acid in Water; Buffer B: 0.075% Formic Acid in Acetonitrile. A 120-min run time is utilized as follow: 0 min – 5% B, 1 to 90 min – 5 to 40%B, 90 to 91 min – 40 to 85% B, maintain at 85% B for 4 min, then back to 5% B in 1 min, finally 24 min re-equilibration at 5%B. Two blanks (1st 100% ACN, 2nd Buffer A) follow each injection to ensure no carry over.

#### MS sequence events

MS is acquired in the Orbitrap using 1 microscan, and a maximum inject time of 900 ms followed by three to 10 data dependant and Multi-Stage Activation (MSA) MS/MS acquisitions for the FT and EN fractions, respectively, in the ion trap (with precursor ions threshold of > 3000); the total cycle time for both MS and MS/MS acquisition is 1.0 s. Peaks targeted for MS/MS fragmentation by collision induced dissociation (CID) or High energy Collision dissociation (HCD) were first isolated with a 2 Da window followed by normalized collision energy of 35%. Dynamic exclusion was activated where former target ions were excluded for 30 s. See below for MS script details on LTQ-Orbitrap parameters used. The data were processed with Progenesis QI proteomics 4.1 (Waters) and protein identification was searched using Mascot search algorithm (version 2.6.2) (Matrix Science). See details below.

#### LF data analyses

Feature extraction, chromatographic/spectral alignment, data filtering, and statistical analysis were performed using Progenesis QI proteomics. First, the .raw data files were imported into the program. A sample run was chosen as a reference (usually at or near the middle of all runs in a set), and all other runs were automatically aligned to that run in order to minimize retention time (RT) variability between runs. No adjustments are necessary in the m/z dimension due to the high mass accuracy of the mass spectrometer (typically < 3 ppm). All runs were selected for detection with an automatic detection limit. Features within RT ranges of 0–16 min and 102–120 min were filtered out, as were features with charge ≥ + 8. A normalization factor was then calculated for each run to account for differences in sample load between injections. The experimental design was setup to group multiple injections from each run. The algorithm then calculates and tabulates raw and normalized abundances, max fold change, and Anova values for each feature in the data set. The features were tagged in sets based on characteristics such as MS/MS > 1, *p* < 0.01, and *p* < 0.01. The MS/MS collected for the experiment were filtered to exclude spectra with rank > 10 or isotope > 3 to ensure that the highest quality MS/MS spectral data are utilized for peptide assignments and subsequent protein ID. The remaining MSMS were exported to an .mgf (Mascot generic file) for database searching (see below). After the Mascot search, an .xml file of the results is created, and then imported into the Progenesis QI proteomics software, where search hits are assigned to corresponding features.

#### Database searching

The .mgf files created by the Progenesis QI proteomics are searched in-house using the Mascot algorithm (Hirosawa et al., 1993, version 2.6.2 for un-interpreted MS/MS spectra. The data was searched against a user specific protein database and also the SWISSPROT Human protein database. Search parameters include: Variable modifications-Carbamidomethyl (Cys), Oxidation (Met), Carbamyl (K) – Note other modification is also used when appropriate (i.e. phosphorylation of S, T, and Y); Peptide mass tolerance - ± 10 ppm; Fragment mass tolerance - ± 0.2 Da; and with Decoy search to get at false discovery rate (FDR). The significance threshold of the ion score was calculated based on a false discovery rate of ≤1%.

Statistical analysis was performed using ANOVA and The Benjamini-Hochberg (BH) method was used to adjust *p* values for multiple-testing false discovery rate. The adjusted *p* ≤ 0.05 was considered as significant. Volcano plot and heatmap was generated using Partek Genomics Suite.

#### SWATH analysis

In order to perform SWATH analysis a relative protein quantification library, consisting of Control and MDD groups was created using CSF samples from this study. Samples were precipitated and trypsin digested overnight using in-solution method and dried using speed vac and resuspended in 20ul of 0.5%TFA and desalted using Millipore C18 ZipTip. Cleaned samples were dried in speed vac and reuspended in 0.1% formic acid for peptide quant using Nanodrop 2000. 1μg of each sample was injected through Eksigent cHiPLC column (75 μm × 15 cm ChromXP C18-CL 3 μm 120 Å) onto 5600 TripleTOF (typical gradient 2–60% ACN in 60 min). CONT and MDD were spiked in HRM calibration peptides for SWATH. CONT and MDD were performed in technical triplicates. Control and MDD pools (each consisting of 3 subjects) were used to create a library of proteins.

To identify proteins present in individual CSF samples, data were analyzed using Protein Pilot search engines against the Swissprot database with the species set as human, specifying trypsin as the enzyme, one missed cleavage, and variable modifications were cysteines as carbamidomethyl and oxidized methionine. Protein Identifications that achieved at least 1% FDR and were identified in all three technical replicates were subjected to further statistical analyses.

The changes in the relative abundance of proteins present in CSF sample were established by comparing the extracted-ion peak intensities of the three technical replicates for each sample. Variation in the relative expression of proteins was assessed by Ztest.

After removal of degraded proteomic samples, nine female (4 MDD and 5 CTRL) and six male (3 MDD AND 3 CTRL) samples that were age and gender matched were used for bioinformatics analysis from the LTQ Orbitrap Elite Mass Spectrometer. Between these two groups, 426 proteins were identified. SWATH analysis identified 307 proteins.

### Ingenuity pathway analysis

Analysis of LTQ Orbitrap Elite mass spectrometry derived proteomics data was performed using Ingenuity Pathways Analysis (IPA) software. The fold expression change data linked to each protein was uploaded as an Excel document to the IPA servers. A core analysis was performed to identify any potentially interesting relationships in the dataset. Overlap with canonical pathways or specific biological functions was calculated algorithmically by the software using its statistical formulas.

### Statistical analyses

Differential expression between major depressive disorder patients was accomplished by performing an ANOVA for each protein and the Benjamini-Hochberg (BH) method was used to adjust *p*-values for multiple testing false discovery rate. An adjusted p-value less than 0.05 was considered significant. The heat map representation of the data was performed using Euclidean minimum distance clustering to determine the similarities of the relative changes. The similarity to biological properties performed by Ingenuity Pathways Analysis was completed with a right-tailed Fisher’s Exact test.

## Results

### Biological functions altered in major depressive disorder (MDD)

**Fig. 2:**
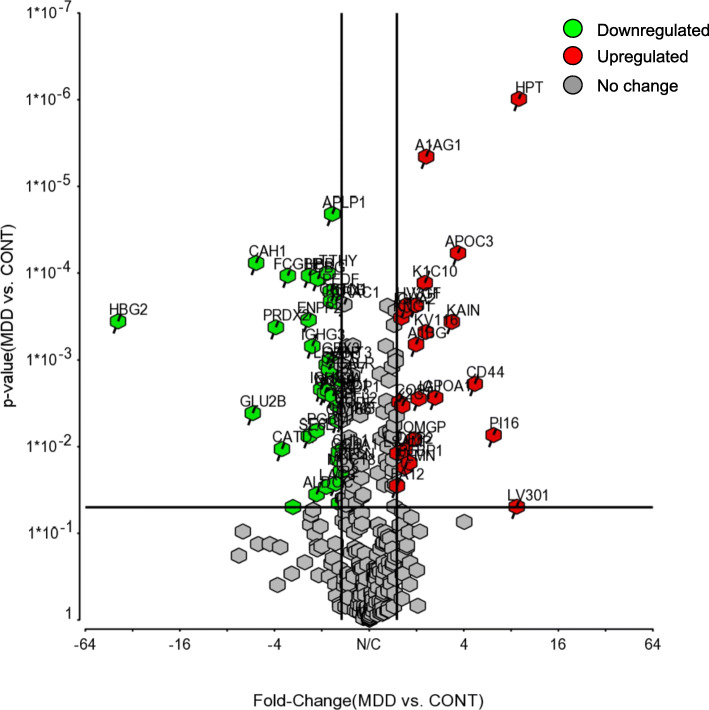
Volcano plot showing the distribution of proteins (307) with relative protein abundance (log2 MDD vs CONT) plotted against its significance level (negative log10 *P*-value), showing significantly (*P* < 0.05) increased (> 1.5; Red) and decreased (< − 1.5; Green) proteins in MDD

**Fig. 3:**
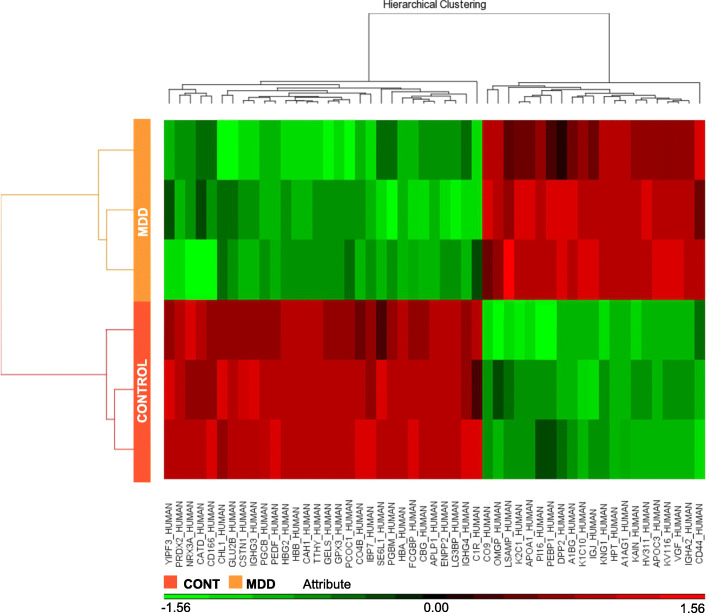
Heat map representation of 6 individual samples abundances for 49 significantly altered proteins after unsupervised hierarchical clustering, segregating samples into CONT (left) and MDD (right) and proteins into up-regulated (bottom) and down-regulated (top) proteins in MDD

After mass spectrometry was completed on the cerebrospinal fluid (CSF) samples, statistical analysis was performed using the Progenesis QI software. This resulted in identifying 43 proteins that were differentially expressed with 23 upregulated and 20 downregulated in MDD. All proteins identified by the LTQ Orbitrap Elite Mass Spectrometer are reported in Supplementary Table 2. Confirmation of proteins was completed with SWATH analysis with 22 proteins being upregulated and 19 downregulated and are reported in Supplementary Table 3. The regulated proteins are shown in Fig. 2 and Fig. 3. Table 1 shows the top ten proteins for both upregulation and downregulation as identified by the LTQ Orbitrap Elite Mass Spectrometer. An Ingenuity Pathway Analysis software core analysis was performed on the complete dataset to elucidate any biological functions related to the dataset. This analysis resulted in the list of disorders/diseases shown in Table 2. This includes inflammatory response, metabolic disease, and organismal injury and abnormalities. Several molecular and cellular functions were also significantly implicated in this dataset. The affected functions listed in Table 3 are cellular compromise, cell-to-cell signaling & interaction, cellular movement, protein synthesis, and cellular development.

**Table 1:** Top ten upregulated and downregulated proteins in MDD cerebrospinal fluid. Columns show UniProt ID, gene symbol, fold change, description, molecular function and cellular localization. Molecular function and cellular localization are from Ingenuity Pathway Analysis (IPA) software

Representative top molecules identified by IPA analysis in MDD patients					
UniProt ID	Gene Symbol	Fold Change (MDD/Ctrl)	Description	Molecular Function	Cellular Localization
Upregulated Genes					
O75460	ERN1	2.26	Ser/thr-protein kinase/endoribonuclease	Kinase	Cytoplasm
Q8IVL0	NAV3	1.96	Neuron navigator 3	Other	Nucleus
P0DOX6		1.88	Immunoglobulin mu heavy chain	Other	Other
Q969Y0	NXPE3	1.67	NXPE family member 3	Other	Other
P01009	SERPINA1	1.65	Alpha-1-antitrypsin	Other	Extracellular Space
P02679	FGG	1.65	Fibrinogen gamma chain	Other	Extracellular Space
P00746	CFD	1.64	Complement factor D	Peptidase	Extracellular Space
O75128	COBL	1.60	Protein cordon-bleu	Other	Plasma Membrane
P51884	LUM	1.59	Lumican	Other	Extracellular Space
P02675	FGB	1.56	Fibrinogen beta chain	Other	Extracellular Space
Downregulated Genes					
P29622	SERPINA4	−2.07	Kallistatin	Other	Extracellular Space
P60174	TPI1	−1.96	Triosephosphate isomerase	Enzyme	Cytoplasm
O43293	DAPK3	−1.65	Death-associated protein kinase 3	Kinase	Cytoplasm
A0A075B6K4	IGLV3–10	−1.65	Immunoglobulin lambda variable 3–10	Other	Other
P04090	RLN2	−1.55	Prorelaxin H2	Other	Extracellular Space
P0C0L5	C4A/C4B	−1.50	Complement C4-B	Other	Extracellular Space
Q8IVW6	ARID3B	−1.49	AT-rich interactive domaincontaining protein 3B	Transcription Regulator	Nucleus
Q6P1S2	C3orf33	−1.45	Protein C3orf33	Other	Extracellular Space
P25705	ATP5F1A	−1.40	ATP synthase subunit alpha, mitochondrial	Transporter	Cytoplasm
P02765	AHSG	−1.39	Alpha-2-HS-glycoprotein	Other	Extracellular Space

**Table 2:** Disorders and diseases identified by Ingenuity Pathway Analysis software as being implicated in MDD. *p*-value ranges were calculated for this dataset for the involvement of including inflammatory response, metabolic disease, and organismal injury and abnormalities. # proteins indicate the number of proteins from this dataset that were implicated as being involved in each of the indicated disorders and diseases

Disorder/Disease	*p*-value Range	# Proteins
**Inflammatory Response**	6.57E-03 - 7.27E-16	25
**Metabolic Disease**	6.51E-03 - 2.42E-09	18
**Organismal Injury and Abnormalities**	7.07E-03 - 2.42E-09	40

**Table 3:** Molecular and cellular functions dysregulated by MDD. Functions include cellular compromise, cell-to-cell signaling & interaction, cellular movement, protein synthesis, and cellular development. # proteins indicate the number of proteins from this dataset that were implicated as being involved in each of the indicated molecular and cellular functions

Molecular and Cellular Functions	*p*-value Range	# Proteins
Cellular Compromise	5.41E-03 - 7.27E-16	20
Cell-To-Cell Signaling & Interaction	6.71E-03 - 4.01E-09	20
Cellular Movement	6.66E-03 - 2.24E-08	24
Protein Synthesis	3.87E-03 - 1.42E-07	16
Cellular Development	5.41E-03 - 3.66E-06	14

### Canonical pathways related to major depressive disorder as generated by IPA

**Fig. 4:**
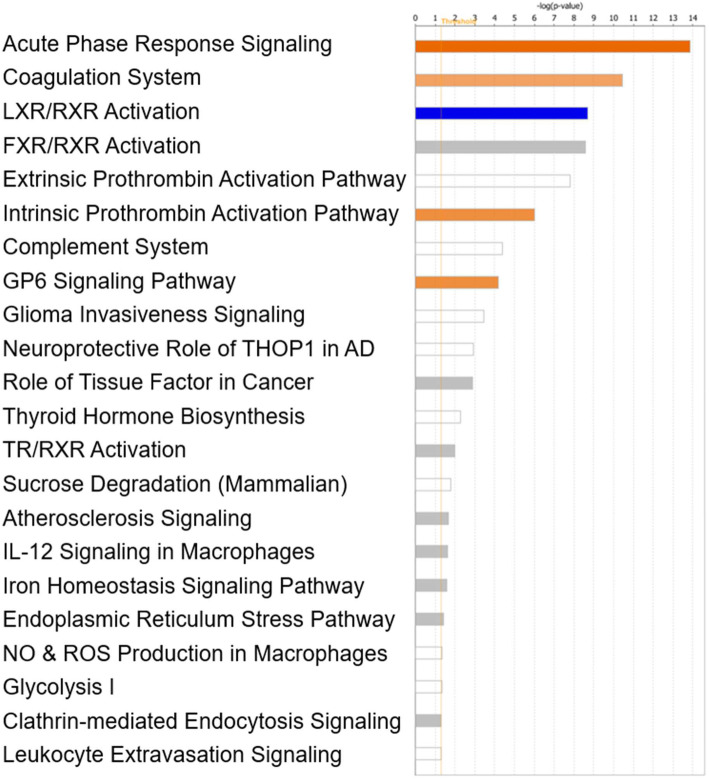
Complete list of canonical pathways associated with the dysregulated proteins identified in this dataset. Y-axis lists the canonical pathway and the x-axis is the log of the corresponding *p*-value for each. Orange coloring indicates the pathway is activated and blue coloring indicates the pathway is inhibited. No coloring indicates insufficient data in the dataset or the IPA knowledge base to determine if the pathway is activated or inhibited

**Fig. 5:**
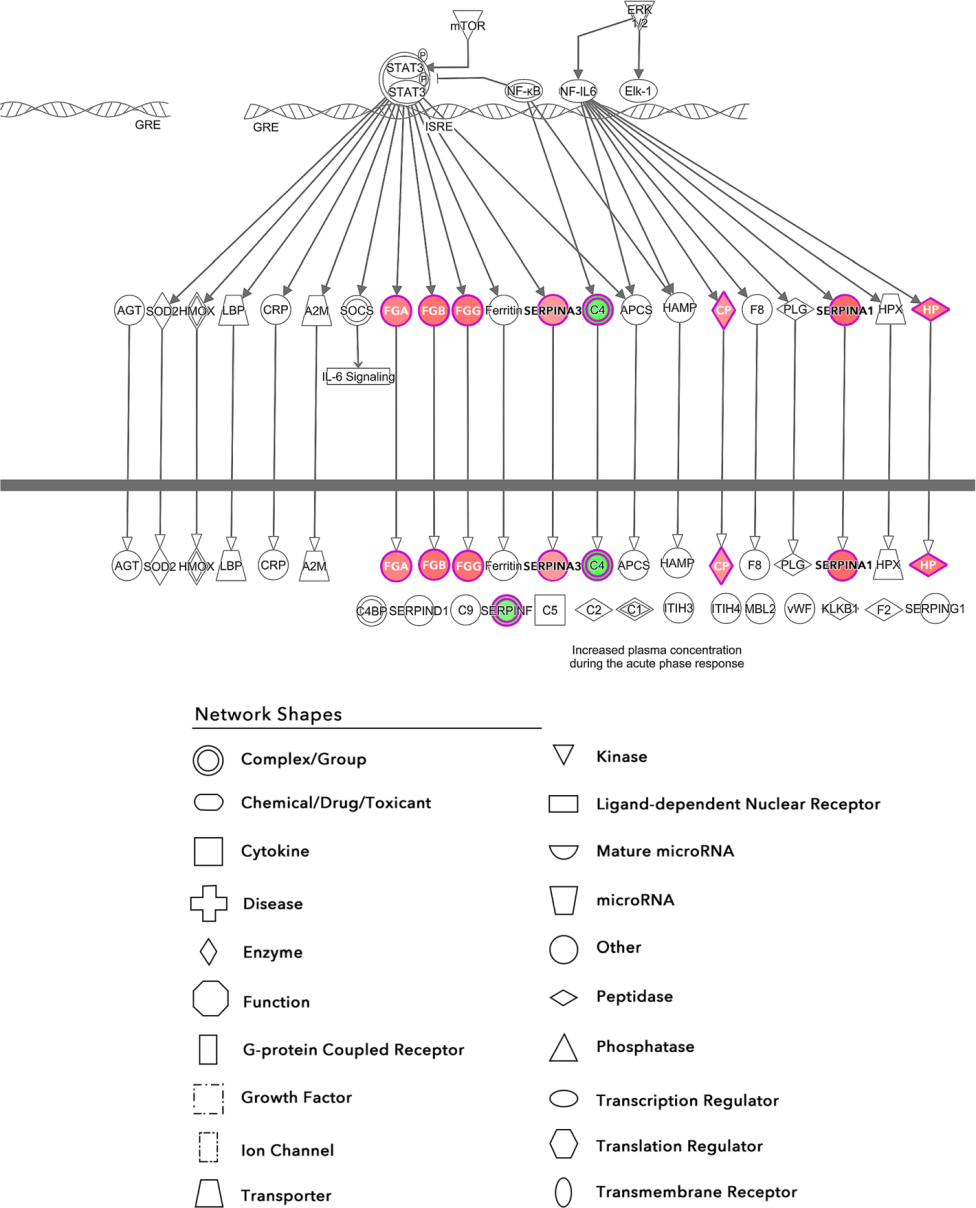
Activation of the acute phase response. Red nodes indicate upregulation and green nodes indicate downregulation. The intensity of the color relates to the extent of regulation with darker meaning greater. An arrow indicates activation whereas a perpendicular line indicates inhibition

The IPA core analysis also identified several canonical pathways that had a substantial overlap with the dataset (Fig. 4). The activated pathways include acute phase response signaling, coagulation system, intrinsic prothrombin activation pathway, and glycoprotein VI (GP6) invasiveness signaling. The sole downregulated pathway was LXR/RXR activation. The most significantly regulated pathway was acute phase response signaling (Fig. 5).

### Upstream regulators generated by IPA software

Shown in Table 4 is a list of upstream regulators that can regulate the processes connected to the dataset. These included cytokines interleukin-6 (IL-6) and oncostatin M (OSM); chemical drugs phenacetin and carboplatin; transcription regulators PR domain zinc finger protein 1 (PRDM1), signal transducer and activator of transcription 3 (STAT3), and PPARG coactivator 1 alpha (PPARGC1A); and the chemical toxicant thioacetamide.

**Table 4:** Upstream regulators with a predicted state of activation or inhibition. Based on the dataset, IPA generated a list of upstream regulators and determined their predicted activation state, activation z-score, and *p*-value of overlap with the dataset

Upstream Regulator	Molecule Type	Predicted Activation State	Activation z-score	*p*-value of overlap
**IL6**	cytokine	Activated	2.84	0.000000104
**phenacetin**	chemical drug	Activated	2	0.00000104
**carboplatin**	chemical drug	Activated	2	0.00000268
**PRDM1**	transcription regulator	Activated	2.433	0.0000037
**thioacetamide**	chemical toxicant	Activated	2.388	0.00000558
**STAT3**	transcription regulator	Activated	2.24	0.00000969
**OSM**	cytokine	Activated	2.594	0.000086
**PPARGC1A**	transcription regulator	Inhibited	−2.204	0.000384

**Fig. 6:**
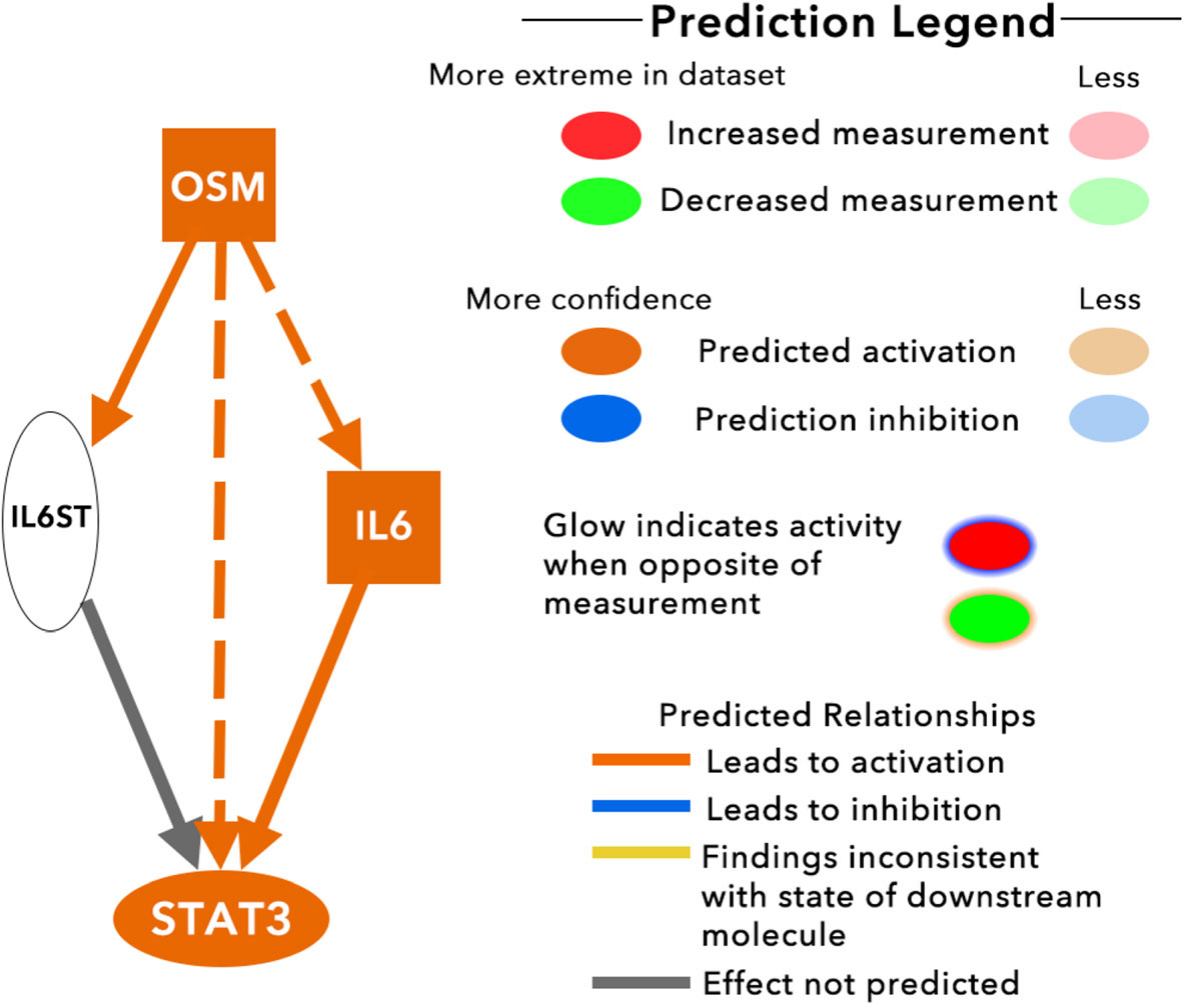
Downstream effects of OSM regulation. Orange nodes denote upregulation, blue nodes denote downregulation, orange arrows denote activation, and blue arrows denote inhibition

Excluding exogenous regulators from this list leaves interleukin-6, oncostatin M, PRDM1, STAT3, and PPARGC1A. As seen in Fig. 6, three of these molecules are interconnected in one pathway leading to the activation of STAT3. This correlates with the data in Fig. 5 as many of the molecules downstream of STAT3 are upregulated.

## Discussion

We performed a proteomic analysis of CSF from MDD and matched non-psychiatric controls and further analyzed the data for functional significance using Ingenuity Pathway Analysis software. This revealed altered molecular and cellular functions, including cellular compromise, cell-to-cell signaling & interaction, cellular movement, protein synthesis, and cellular development. Disease/disorder processes related to MDD were also statistically significant, including inflammatory response, metabolic disease, and organismal injury and abnormalities.

Previous research has shown that MDD patients have elevated levels of inflammatory proteins including those revealed in our study such as alpha-1-antitrypsin [17, 18]. The role of inflammation in depression has attracted significant attention and there is substantial evidence to indicate that it is important to disease pathophysiology. Studies have described how a western diet that leads to adiposity also increases the pro-inflammatory state of the body and correlates with depressive symptoms [19]. Another study investigated how core MDD symptoms such as exaggerated response to negative information, altered reward processing, and decreased cognitive control correlate strongly with inflammation [20]. Several chemokines are dysregulated in the blood of depressive patients [21], including elevated C-Reactive Protein (CRP) levels [22, 23]. The above information insinuates a correlational role of adiposity independent or dependent inflammation with depressive symptoms.

It is interesting to note that the reward pathway is strongly influenced by inflammatory cytokines such as interferons, interleukin-1β, and tumor necrosis factor [24]. This modulation of the reward pathway by pro-inflammatory signaling could emerge as a causal link between our proteomics data and disease phenotype with the decreased reward pathway leading to the anhedonia that is common to the disorder [25]. The pro-inflammatory state may also lead to MDD through direct neurotoxicity of brain regions involved in emotional regulation including the hippocampus, amygdala, and anterior cingulate cortex. This neurotoxicity is potentially mediated by NMDA receptor excitotoxicity, reactive oxygen and nitrogen species, and reactive gliosis [26]. The adverse impact on these brain structures could influence the cortico-striatal pathway as both the hippocampus and amygdala have inputs to the striatum, and the cortex has many bidirectional relationships with the thalamus and striatum [27]. In addition to highlighting the acute phase response signaling system, our dataset also implicated several upstream regulators that could have a role in depression. These signaling molecules are oncostatin M (OSM), interleukin 6, and STAT3. IL-6 and STAT3 have been previously shown to be involved in serotonin transporter function and depression-like behavior [28]. OSM has been shown to have various effects in the body including inflammation, but it has not been well studied with regard to depression [29]. Clinical studies (NCT00291239; NCT03080025) are investigating the role of IL-6 as a biomarker or causative molecule in depression, but none are investigating STAT3 or oncostatin M. It should be noted that STAT3 is activated by elevated IL-6 and oncostatin M belongs to the IL-6 family. Future research focused on manipulating levels of these molecules in preclinical models can shed light on whether they play direct roles in modulating depressive behavior.

Downregulated proteins found in our study and in the literature include energy metabolism proteins such as triosephosphate isomerase [30, 31]. Studies examining the comorbidity of depression and metabolic dysregulation have been supported by reports that have focused on poor glycemic control [32], diabetes [33], and metabolic syndrome [34]. The specific link between these conditions has not been sufficiently elucidated. Also, the directionality of the relationship is still being debated. It is clear that there is a correlation between hyperglycemia and depression [32]. Importantly, in patients with Type II diabetes the psychosocial stress or a biochemical change as a result of the treatment does not cause any alteration in the rate of depression which suggests that an alternate variable must be involved. Adiposity leads to a heightened inflammation state in the body [35] and this could affect the brain leading to increased vulnerability for major depressive disorder. An interesting avenue of future research is understanding the links between hyperglycemia, adiposity, inflammation, and major depressive disorder.

Lastly, fibrinogen has been shown in previous studies to be positively correlated with MDD [36, 37]. In patients with high CSF levels of fibrinogen, significant white matter tract abnormalities were observed [38]. Haptoglobin has also been implicated in MDD over the past few decades [18] and more recent research has focused on investigating the effects of different haptoglobin genotypes [39, 40]. These findings indicate a potential vulnerability of the BBB in depression and is worthy of further investigation. Our results provide additional support that these proteins are involved in MDD.

## Conclusion

The proteome profiling data in this report identified several potential biological functions that may be disrupted as part of the pathophysiology of MDD. These include inflammatory response, metabolic disease, and organismal injury/abnormalities. Additionally, several biological functions including cellular compromise, cell-to-cell signaling and interaction, cellular movement, protein synthesis, and cellular development were also suggested to be involved in MDD. Acute phase response was identified as a significantly impacted canonical pathway by this analysis. Finally, several endogenous upstream regulators including interleukin 6, oncostatin M, STAT3, PRDM1, and PPARGC1A were identified by statistical analyses of the proteome profiling data.

## Supplementary information

**Additional file 1: Supplementary Table 1.** A demographic table of patient data including gender and major depressive disorder status.

**Additional file 2: Supplementary Table 2.** A complete list of all proteins identified by LTQ Orbitrap Elite Mass Spectrometer. Green highlighted ANOVAs are statistically significant.

**Additional file 3: Supplementary Table 3.** A complete list of all proteins identified by SWATH analysis.

**Additional file 4: Supplementary Figure 1.** Complete acute phase response signaling. Red nodes are upregulated and green nodes are down regulated.

## Data Availability

Data may be found in supplementary figures and on Mendeley (Franzen, Avery; Sathyanesan, Samuel; Duman, Ronald; Williams, Kenneth; Nairn, Angus; Lam, Tukiet; Carpenter, Linda; Kumar, Vikas (2020), “Proteomic Analysis of Cerebrospinal Fluid in Major Depressive Disorder”, Mendeley Data, v1, 10.17632/th4h8988d4.1).

## Abbreviations

ACN: Acetonitrile
AGC: Automatic gain control
ANOVA: Analysis of variance
BH: Benjamini-hochberg
CARTPT: Cocaine and amphetamine regulated transcript
CONT: Control
CRP: C-reactive protein
CSF: Cerebrospinal fluid
CTRL: Control
DSM-IV: Diagnostic and statistical manual of mental disorders-IV
FA: Formic acid
GRIA4: Glutamate ionotropic receptor AMPA type subunit 4
GWAS: Genome wide association study
HCD: Higher-energy collisional dissociation
HPA Axis: Hypothalamic pituitary adrenal axis
IFN: Interferon
IL: Interleukin
IPA: Ingenuity pathway analysis
IRB: Institutional review board
LFQ: Label free quantitation
LP: Lumbar puncture
LXR/RXR: Liver X receptor-retinoid X receptor
MDD: Major depressive disorder
MS: Mass spectrometry
NIMH: National institute of mental health
NMDA: N-methyl- d-aspartate
NPTXR: Neuronal pentraxin receptor
NRXN3: Neurexin 3
OSM: Oncostatin M
PCSSK1N: Proprotein convertase subtilisin/kexin type 1 inhibitor
PPARGC1A: Peroxisome proliferator-activated receptor gamma coactivator 1-alpha
PRDM1: PR domain zinc finger protein 1
STAT3: Signal transducer and activator of transcription 3
SWATH: Sequential windowed acquisition of all theoretical fragment ion mass spectra
TNF: Tumor necrosis factor
TNF-α: Tumor necrosis factor-alpha
VGF: Non acronymic
