# Supplementary material for: Cerebrospinal fluid proteome evaluation in major depressive disorder by mass spectrometry
Source: BMC Psychiatry. 2020 Oct 1;20:481. doi: 10.1186/s12888-020-02874-9 (PMC7528485; doi:10.1186/s12888-020-02874-9)
Supplement: Supplementary file 1 — Additional file 1: Supplementary Table 1. A demographic table of patient data including gender and major depressive disorder status. [file 12888_2020_2874_MOESM1_ESM.pdf]

	MDD	Non-psychiatric control	Age	Scale	Total
Male	3	3	27-53	HDRS > 17	6
Female	4	5	19-54	HDRS > 17	9
Total	7	8			15
