# Supplementary material for: Cerebrospinal fluid proteome evaluation in major depressive disorder by mass spectrometry
Source: BMC Psychiatry. 2020 Oct 1;20:481. doi: 10.1186/s12888-020-02874-9 (PMC7528485; doi:10.1186/s12888-020-02874-9)
Supplement: Supplementary file 2 — Additional file 2: Supplementary Table 2. A complete list of all proteins identified by LTQ Orbitrap Elite Mass Spectrometer. Green highlighted ANOVAs are statistically significant. [file 12888_2020_2874_MOESM2_ESM.pdf]

Accession	Peptides	Score	Anova (p)*	Fold
sp|P02768|ALBU_HUMAN	92 (91)	9231.92	0.09	1.17
sp|P01024|CO3_HUMAN	64 (61)	5240.22	0.38	1.04
sp|P02787|TRFE_HUMAN	45 (44)	4406.24	0.9	1.01
sp|P0C0L5|CO4B_HUMAN	37 (37)	3107.14	2.97E-03	1.5
sp|P01009|A1AT_HUMAN	29 (27)	2666.8	4.95E-05	1.65
sp|P01023|A2MG_HUMAN	35 (35)	2577.72	0.06	1.24
sp|P02751|FINC_HUMAN	28 (27)	1851.25	0.46	1.11
sp|P10909|CLUS_HUMAN	22 (21)	1779.94	0.13	1.25
sp|P02774|VTDB_HUMAN	25 (24)	1778.5	0.16	1.19
sp|P02649|APOE_HUMAN	23 (23)	1660.39	0.67	1.12
sp|P05060|SCG1_HUMAN	19 (18)	1592.96	0.45	1.17
sp|O00533|NCHL1_HUMAN	22 (21)	1552.74	0.62	1.14
sp|P02647|APOA1_HUMAN	16 (16)	1507.2	0.69	1.06
sp|P06396|GELS_HUMAN	19 (19)	1443.24	0.8	1.01
sp|P00738|HPT_HUMAN	21 (20)	1418.67	0.05	1.47
sp|Q14515|SPRL1_HUMAN	18 (17)	1397.95	0.69	1.09
sp|P02766|TTHY_HUMAN	12 (12)	1372.1	0.18	1.21
sp|P00450|CERU_HUMAN	19 (19)	1324.41	0.03	1.19
sp|P01008|ANT3_HUMAN	18 (18)	1307.66	0.03	1.28
sp|P05067|A4_HUMAN	15 (13)	1280.28	0.91	1.01
sp|P0DOX5|IGG1_HUMAN	13 (5)	1271.93	0.72	1.07

sp|Q13822|ENPP2_HUMAN	21 (21)	1219.68	0.99	1.01
sp|P02790|HEMO_HUMAN	15 (15)	1195.78	0.06	1.2
sp|P06727|APOA4_HUMAN	14 (14)	1189.16	0.25	1.13
sp|Q92823|NRCAM_HUMAN	17 (17)	1150.95	0.32	1.25
sp|P01019|ANGT_HUMAN	11 (11)	1146.84	0.05	1.33
sp|P01860|IGHG3_HUMAN	13 (5)	1105.44	0.23	1.16
sp|P23142|FBLN1_HUMAN	15 (15)	1102.13	0.48	1.1
sp|P41222|PTGDS_HUMAN	12 (12)	1067.94	0.92	1
sp|P36955|PEDF_HUMAN	14 (12)	1066.91	0.61	1.05
sp|P68871|HBB_HUMAN	11 (11)	1060.85	0.25	1.54
sp|Q9UBP4|DKK3_HUMAN	12 (11)	1055.67	0.53	1.15
sp|P10645|CMGA_HUMAN	10 (10)	1024.71	0.34	1.36
sp|P08603|CFAH_HUMAN	16 (16)	993.06	0.3	1.09
sp|O94985|CSTN1_HUMAN	14 (14)	960.16	0.62	1.12
sp|Q92876|KLK6_HUMAN	10 (10)	958.42	0.42	1.12
sp|O15240|VGF_HUMAN	13 (12)	941.95	0.3	1.25
sp|P51693|APLP1_HUMAN	12 (12)	932.65	0.6	1.08
sp|P02763|A1AG1_HUMAN	9 (7)	883.11	0.78	1.02
sp|P19652|A1AG2_HUMAN	9 (7)	872.57	7.47E-03	1.28
sp|P01876|IGHA1_HUMAN	12 (8)	857.4	0.61	1.29
sp|Q8WXD2|SCG3_HUMAN	13 (13)	852.16	0.65	1.1
sp|Q96KN2|CNDP1_HUMAN	12 (11)	850.22	0.08	1.25

sp|P00734|THRB_HUMAN	11 (11)	835.09	0.55	1.04
sp|P01859|IGHG2_HUMAN	11 (4)	815.7	0.2	1.3
sp|P69905|HBA_HUMAN	10 (10)	806.26	0.39	1.6
sp|P00751|CFAB_HUMAN	13 (12)	775.44	0.23	1.08
sp|P0DOX7|IGK_HUMAN	6 (3)	767.63	0.2	1.21
sp|Q12805|FBLN3_HUMAN	11 (11)	765.47	0.48	1.16
sp|P13591|NCAM1_HUMAN	12 (12)	760.96	0.39	1.18
sp|P01034|CYTC_HUMAN	8 (8)	757.77	0.37	1.21
sp|P02765|FETUA_HUMAN	7 (7)	755.67	4.34E-03	1.39
sp|P01834|IGKC_HUMAN	4 (1)	702.52	0.76	1.03
sp|P00747|PLMN_HUMAN	13 (13)	676.09	0.06	1.21
sp|Q12860|CNTN1_HUMAN	12 (12)	669.12	0.83	1.04
sp|Q16270|IBP7_HUMAN	8 (8)	664.73	0.18	1.13
sp|P04196|HRG_HUMAN	9 (9)	653.26	0.74	1.02
sp|Q92520|FAM3C_HUMAN	9 (9)	614.09	0.59	1.06
sp|P13645|K1C10_HUMAN	8 (7)	610.35	0.21	1.9
sp|P0DOY2|IGLC2_HUMAN	6 (2)	604.93	0.67	1.03
sp|B9A064|IGLL5_HUMAN	6 (2)	603.33	0.98	1.03
sp|P01042|KNG1_HUMAN	10 (10)	600.53	0.07	1.15
sp|P05155|IC1_HUMAN	8 (8)	588.49	0.77	1
sp|P01861|IGHG4_HUMAN	8 (2)	581.84	0.59	1.06
sp|P02679|FIBG_HUMAN	8 (6)	580.32	5.53E-03	1.65

sp|P01011|AACT_HUMAN	8 (8)	577.92	0.03	1.13
sp|Q99435|NELL2_HUMAN	9 (9)	559.43	0.09	1.32
sp|P20774|MIME_HUMAN	9 (9)	538.88	0.79	1.01
sp|P04264|K2C1_HUMAN	9 (5)	538.56	0.11	2.11
sp|Q08380|LG3BP_HUMAN	6 (6)	535.31	0.91	1.01
sp|Q9UHG2|PCSK1_HUMAN	8 (8)	528.43	0.66	1.06
sp|Q14624|ITIH4_HUMAN	9 (9)	509.52	0.83	1.02
sp|P10643|CO7_HUMAN	9 (9)	508.17	0.93	1
sp|P04217|A1BG_HUMAN	7 (7)	507.66	0.14	1.14
sp|P02675|FIBB_HUMAN	7 (7)	496.07	3.81E-03	1.56
sp|P18065|IBP2_HUMAN	7 (7)	464.84	0.81	1.02
sp|P07339|CATD_HUMAN	7 (7)	454.27	0.02	1.2
sp|O43505|B4GA1_HUMAN	7 (7)	453.18	0.27	1.24
sp|P35527|K1C9_HUMAN	6 (4)	443.8	0.12	1.52
sp|Q15818|NPTX1_HUMAN	5 (5)	442.67	0.73	1.06
sp|P10451|OSTP_HUMAN	6 (6)	436.43	0.67	1.13
sp|P02671|FIBA_HUMAN	6 (6)	435.93	3.64E-03	1.38
sp|P02652|APOA2_HUMAN	6 (6)	433.02	0.08	1.3
sp|P08571|CD14_HUMAN	7 (7)	428.42	0.12	1.24
sp|P43652|AFAM_HUMAN	8 (8)	425.37	0.41	1.08
sp|P02749|APOH_HUMAN	7 (7)	424.53	0.06	1.49
sp|P07602|SAP_HUMAN	7 (7)	418.87	0.48	1.18

sp|O95502|NPTXR_HUMAN	5 (5)	402.19	0.52	1.04
sp|P13521|SCG2_HUMAN	8 (7)	398.98	0.62	1.21
sp|P0DOX2|IGA2_HUMAN	6 (1)	379.55	0.41	1.02
sp|P19823|ITIH2_HUMAN	6 (6)	365.91	0.8	1.05
sp|P00441|SODC_HUMAN	4 (4)	359.73	0.56	1.12
sp|P55290|CAD13_HUMAN	5 (5)	354.4	0.13	1.32
sp|P61769|B2MG_HUMAN	3 (3)	351.73	0.53	1.12
sp|P02750|A2GL_HUMAN	6 (6)	342.42	0.02	1.26
sp|P05090|APOD_HUMAN	5 (5)	331.16	0.9	1.04
sp|P05156|CFAI_HUMAN	5 (5)	330.82	0.77	1.02
sp|P09871|C1S_HUMAN	6 (6)	327.11	0.42	1.12
sp|P17900|SAP3_HUMAN	4 (4)	324.99	0.25	1.21
sp|P05546|HEP2_HUMAN	8 (8)	324.02	0.7	1.03
sp|P05452|TETN_HUMAN	3 (3)	322.93	0.86	1.2
sp|Q9Y6R7|FCGBP_HUMAN	7 (7)	321.64	0.79	1.47
sp|P01877|IGHA2_HUMAN	5 (0)	321.35	---	---
sp|P36222|CH3L1_HUMAN	6 (6)	314.88	0.03	1.38
sp|P12109|CO6A1_HUMAN	4 (4)	311.84	0.62	1.09
sp|P35908|K22E_HUMAN	4 (2)	308.2	0.28	1.3
sp|P61916|NPC2_HUMAN	5 (5)	301.06	0.72	1.05
sp|P08697|A2AP_HUMAN	5 (5)	288.05	0.04	1.28
sp|P07998|RNAS1_HUMAN	5 (5)	287.98	0.89	1.03

sp|P08294|SODE_HUMAN	3 (3)	285.02	0.97	1.01
sp|P02748|CO9_HUMAN	5 (5)	276.24	0.53	1.04
sp|P30086|PEBP1_HUMAN	5 (5)	275.9	0.39	1.13
sp|P09486|SPRC_HUMAN	5 (5)	273.24	0.88	1.02
sp|P0DOX6|IGM_HUMAN	3 (3)	272.38	5.65E-03	1.88
sp|P78324|SHPS1_HUMAN	6 (6)	269.09	0.2	1.25
sp|Q02246|CNTN2_HUMAN	5 (5)	268.36	0.7	1.01
sp|Q14118|DAG1_HUMAN	4 (4)	267.06	0.44	1.23
sp|P24592|IBP6_HUMAN	3 (3)	264.14	0.89	1.02
sp|P01210|PENK_HUMAN	3 (3)	261.21	0.94	1
sp|Q96GW7|PGCB_HUMAN	5 (4)	260.31	0.14	1.3
sp|Q9P121|NTRI_HUMAN	4 (4)	237.78	0.54	1.19
sp|Q16610|ECM1_HUMAN	4 (4)	237.23	0.27	1.28
sp|P14618|KPYM_HUMAN	4 (4)	230.36	0.75	1.06
sp|P04004|VTNC_HUMAN	3 (3)	227.79	0.02	1.21
sp|P02747|C1QC_HUMAN	4 (4)	225.83	0.1	1.26
sp|Q06481|APLP2_HUMAN	4 (3)	225.69	0.64	1.08
sp|P19021|AMD_HUMAN	3 (3)	218.14	0.8	1.07
sp|P04216|THY1_HUMAN	2 (2)	217.53	0.52	1.29
sp|Q9NQ79|CRAC1_HUMAN	4 (4)	214.08	0.89	1.04
sp|A0A075B6K5|LV39_HUMAN	2 (2)	210.79	0.46	1.13
sp|P05408|7B2_HUMAN	5 (5)	205.24	0.51	1.14

sp|P19022|CADH2_HUMAN	3 (3)	202.08	0.44	1.21
sp|Q96FE7|P3IP1_HUMAN	3 (3)	195.82	0.58	1.1
sp|P02746|C1QB_HUMAN	3 (3)	187.53	0.04	1.23
sp|O14594|NCAN_HUMAN	4 (3)	183.28	0.86	1.02
sp|P41271|NBL1_HUMAN	2 (2)	182.6	0.49	1.15
sp|P02760|AMBP_HUMAN	3 (3)	179.88	0.75	1.02
sp|P04156|PRIO_HUMAN	3 (3)	172.48	0.55	1.03
sp|O75711|SCRG1_HUMAN	3 (3)	171.1	0.87	1.04
sp|P00736|C1R_HUMAN	3 (3)	170.83	0.91	1
sp|Q13449|LSAMP_HUMAN	3 (3)	169.11	0.43	1.22
sp|P62979|RS27A_HUMAN	2 (2)	165.92	0.61	1.03
sp|O15394|NCAM2_HUMAN	2 (2)	161.26	0.44	1.26
sp|Q96PD5|PGRP2_HUMAN	3 (3)	158.6	0.12	1.26
sp|P13987|CD59_HUMAN	2 (2)	155.55	0.92	1.11
sp|P02753|RET4_HUMAN	3 (3)	154.08	0.2	1.2
sp|P08185|CBG_HUMAN	3 (3)	143.05	0.22	1.11
sp|P06681|CO2_HUMAN	2 (1)	139.45	0.99	1.04
sp|P0DP03|HV335_HUMAN	2 (1)	136.37	0.59	1.05
sp|O60888|CUTA_HUMAN	3 (3)	135.93	0.72	1.1
sp|Q15582|BGH3_HUMAN	2 (2)	133.33	0.76	1.04
sp|P16035|TIMP2_HUMAN	2 (1)	129.59	0.02	1.18
sp|P54289|CA2D1_HUMAN	2 (2)	129.55	0.37	1.16

sp|P16870|CBPE_HUMAN	3 (3)	129.18	0.6	1.05
sp|O75326|SEM7A_HUMAN	3 (3)	127.55	0.2	1.21
sp|Q15904|VAS1_HUMAN	1 (1)	125.47	0.91	1.11
sp|Q8TCZ2|C99L2_HUMAN	2 (2)	123.78	0.42	1.25
sp|Q8IWU5|SULF2_HUMAN	1 (1)	118.71	0.6	1.1
sp|Q8N126|CADM3_HUMAN	2 (2)	114.12	0.96	1
sp|Q9BY67|CADM1_HUMAN	1 (1)	112.45	0.8	1.08
sp|Q9NQX5|NPDC1_HUMAN	1 (1)	108.78	0.74	1.11
sp|O95196|CSPG5_HUMAN	1 (1)	107.83	0.42	1.09
sp|A0A0B4J1X5|HV374_HUMAN	2 (1)	106.6	0.68	1.12
sp|P01619|KV320_HUMAN	2 (2)	106.57	0.05	1.25
sp|Q15113|PCOC1_HUMAN	2 (2)	101.56	0.16	1.15
sp|P78509|RELN_HUMAN	2 (2)	95.81	0.62	1.11
sp|O94856|NFASC_HUMAN	2 (1)	95.33	0.74	1.06
sp|P02656|APOC3_HUMAN	1 (1)	94.17	0.63	1.03
sp|Q8TAG5|VTM2A_HUMAN	2 (2)	90.64	0.01	1.28
sp|P23471|PTPRZ_HUMAN	2 (2)	90.32	0.2	1.24
sp|P43121|MUC18_HUMAN	1 (1)	89.65	0.77	1.21
sp|P02745|C1QA_HUMAN	2 (2)	89.32	0.36	1.1
sp|P01714|LV319_HUMAN	2 (2)	89.11	0.66	1.06
sp|A0A0C4DH55|KVD07_HUMAN	2 (2)	87.94	0.45	1.1

sp|A0A075B6S2|KVD29_HUMAN	1 (1)	86.38	0.4	2.22
sp|Q92932|PTPR2_HUMAN	1 (1)	86.13	0.59	1.18
sp|P07333|CSF1R_HUMAN	1 (1)	85.58	0.31	1.2
sp|P01701|LV151_HUMAN	2 (2)	83.06	0.29	1.77
sp|Q5SYB0|FRPD1_HUMAN	2 (1)	81.65	0.57	1.05
sp|P17174|AATC_HUMAN	1 (1)	80.4	0.79	1.08
sp|P48058|GRIA4_HUMAN	1 (1)	80.26	0.18	1.9
sp|Q92859|NEO1_HUMAN	2 (2)	80.01	0.09	1.35
sp|P01700|LV147_HUMAN	1 (1)	78.69	0.9	1.02
sp|P58400|NRX1B_HUMAN	1 (1)	75.45	0.19	1.33
sp|P01303|NPY_HUMAN	1 (1)	75.14	0.92	1.08
sp|P04406|G3P_HUMAN	1 (1)	72.7	0.37	1.14
sp|P61278|SMS_HUMAN	1 (1)	72.66	0.72	1.42
sp|Q8NFZ8|CADM4_HUMAN	2 (2)	71.22	0.36	1.33
sp|Q9HDB5|NRX3B_HUMAN	1 (1)	68.45	0.38	1.13
sp|Q9NUQ6|SPS2L_HUMAN	1 (1)	68.4	0.72	1.07
sp|P07225|PROS_HUMAN	1 (1)	68.17	0.99	1.03
sp|Q13332|PTPRS_HUMAN	1 (1)	67.94	0.23	1.23
sp|P08123|CO1A2_HUMAN	1 (1)	67.35	0.53	1.13
sp|P29622|KAIN_HUMAN	1 (1)	65.77	7.15E-03	2.07
sp|Q02818|NUCB1_HUMAN	1 (1)	65.03	0.59	1.04
sp|P43251|BTD_HUMAN	1 (1)	62.9	0.69	1.05

sp|Q9GZM5|YIPF3_HUMAN	2 (2)	62.75	0.4	1.11
sp|P23284|PPIB_HUMAN	1 (1)	62.11	0.63	1.04
sp|P55058|PLTP_HUMAN	1 (1)	62.06	0.16	1.17
sp|P05543|THBG_HUMAN	2 (2)	61.81	0.75	1.05
sp|P51884|LUM_HUMAN	1 (1)	61.18	0.02	1.59
sp|A0A0B4J1V1|HV321_HUMAN	1 (0)	60.64	---	---
sp|Q7Z3B1|NEGR1_HUMAN	1 (1)	60.53	0.12	1.35
sp|Q16568|CART_HUMAN	1 (1)	60.42	0.25	1.31
sp|P04075|ALDOA_HUMAN	2 (2)	59.26	0.37	1.23
sp|Q6PGP7|TTC37_HUMAN	2 (0)	59.14	---	---
sp|P16070|CD44_HUMAN	1 (1)	58.76	0.04	1.51
sp|P35542|SAA4_HUMAN	1 (0)	58.75	---	---
sp|Q9BQT9|CSTN3_HUMAN	1 (1)	58.66	0.31	1.52
sp|Q99969|RARR2_HUMAN	1 (1)	58.37	0.85	1.09
sp|P13473|LAMP2_HUMAN	1 (1)	57.21	0.15	1.42
sp|Q4G0X9|CCD40_HUMAN	2 (2)	57	0.74	1.11
sp|Q9NT99|LRC4B_HUMAN	1 (1)	56.06	0.77	1.08
sp|Q96B26|EXOS8_HUMAN	2 (0)	54.93	---	---
sp|Q14982|OPCM_HUMAN	1 (1)	54.93	0.61	1.21
sp|Q66K66|TM198_HUMAN	2 (2)	53.49	0.59	1.12
sp|O75509|TNR21_HUMAN	1 (1)	53.44	0.13	1.21

sp|Q16769|QPCT_HUMAN	1 (1)	52.18	0.22	2.34
sp|P25786|PSA1_HUMAN	2 (0)	52.18	---	---
sp|P00748|FA12_HUMAN	1 (1)	51.86	0.56	1.07
sp|P07711|CATL1_HUMAN	1 (1)	51.86	0.93	1
sp|P02654|APOC1_HUMAN	1 (1)	51.11	0.33	1.1
sp|Q6P1S2|CC033_HUMAN	2 (1)	50.9	0.03	1.45
sp|Q8WZ42|TITIN_HUMAN	2 (1)	50.35	0.28	1.14
sp|P06312|KV401_HUMAN	1 (1)	49.16	0.41	1.11
sp|Q93070|NAR4_HUMAN	2 (0)	48.71	---	---
sp|P13671|CO6_HUMAN	1 (1)	48.61	0.17	1.7
sp|Q8TBY8|PMFBP_HUMAN	1 (1)	45.79	0.2	1.13
sp|Q9P0K1|ADA22_HUMAN	1 (1)	45.42	0.97	1.19
sp|Q8NGF8|OR4B1_HUMAN	1 (1)	45.23	0.92	1.02
sp|Q9C091|GRB1L_HUMAN	2 (1)	44.03	0.15	1.19
sp|P00746|CFAD_HUMAN	1 (1)	42.95	1.22E-03	1.64
sp|P07360|CO8G_HUMAN	1 (1)	41.79	0.12	1.16
sp|O15078|CE290_HUMAN	1 (0)	41.64	---	---
sp|Q9H211|CDT1_HUMAN	1 (0)	41.52	---	---
sp|P53367|ARFP1_HUMAN	1 (0)	41.46	---	---
sp|Q5VU69|CA189_HUMAN	1 (1)	41.27	0.27	1.55
sp|P06307|CCKN_HUMAN	1 (1)	40.94	0.71	1.06
sp|Q8NHQ9|DDX55_HUMAN	1 (1)	40.11	0.2	1.13

sp|Q8IV63|VRK3_HUMAN	1 (1)	39.93	0.26	1.18
sp|Q9UBQ6|EXTL2_HUMAN	1 (1)	39.5	0.67	1.07
sp|P39060|COIA1_HUMAN	1 (1)	39.32	0.83	1.06
sp|Q96M02|CJ090_HUMAN	1 (0)	37.44	---	---
sp|Q96T17|MA7D2_HUMAN	1 (1)	36.39	0.63	1.05
sp|Q8WYP5|ELYS_HUMAN	1 (1)	36.15	0.92	1.12
sp|Q5SY16|NOL9_HUMAN	1 (1)	35.86	0.93	1.02
sp|A0A0A0MT89|KJ01_HUMAN	1 (1)	35.7	0.18	3.55
sp|Q6UB99|ANR11_HUMAN	1 (0)	35.36	---	---
sp|Q9P2S2|NRX2A_HUMAN	1 (1)	35.26	0.04	1.44
sp|P25705|ATPA_HUMAN	1 (1)	35.2	6.87E-04	1.4
sp|P0DP23|CALM1_HUMAN	1 (1)	35.16	0.83	1.01
sp|A0A0U1RQE8|GLYLB_HUMAN	1 (1)	34.84	0.22	1.09
sp|Q9Y6Y8|S23IP_HUMAN	1 (1)	34.69	0.22	1.33
sp|Q9H3P7|GCP60_HUMAN	1 (1)	34.35	0.13	1.29
sp|Q14533|KRT81_HUMAN	1 (1)	34.22	0.61	1.09
sp|P55786|PSA_HUMAN	1 (0)	33.87	---	---
sp|P27348|1433T_HUMAN	1 (0)	33.62	---	---
sp|Q9NUV7|SPTC3_HUMAN	1 (1)	33.51	0.26	1.17
sp|O00294|TULP1_HUMAN	1 (1)	33.5	0.09	1.5
sp|P25311|ZA2G_HUMAN	1 (1)	33.43	0.08	1.43
sp|Q5TF58|IFFO2_HUMAN	1 (1)	33.36	0.89	1.47

sp|Q03426|KIME_HUMAN	1 (1)	32.86	0.53	1.1
sp|Q8IXQ8|PDZD9_HUMAN	1 (1)	32.81	0.66	1.03
sp|Q3KNS1|PTHD3_HUMAN	1 (1)	32.66	0.42	1.11
sp|Q96SZ4|ZSC10_HUMAN	1 (0)	32.53	---	---
sp|Q9P0Z9|SOX_HUMAN	1 (1)	32.37	0.05	1.29
sp|Q68CJ9|CR3L3_HUMAN	1 (1)	32.31	0.06	1.88
sp|P15814|IGLL1_HUMAN	1 (1)	32.26	0.64	1.08
sp|P23515|OMGP_HUMAN	1 (1)	32.24	0.16	1.89
sp|P0DP58|LYNX1_HUMAN	1 (1)	32.06	0.45	1.36
sp|B1AJZ9|FHAD1_HUMAN	1 (1)	32.03	0.08	1.93
sp|P12259|FA5_HUMAN	1 (0)	32.02	---	---
sp|O60879|DIAP2_HUMAN	1 (1)	31.83	0.94	1.01
sp|Q9BXJ9|NAA15_HUMAN	1 (0)	31.55	---	---
sp|A0A0B4J1V0|HV315_HUMAN	1 (1)	31.5	0.39	1.12
sp|P43405|KSYK_HUMAN	1 (0)	31.37	---	---
sp|Q96QV1|HHIP_HUMAN	1 (0)	31.36	---	---
sp|O75460|ERN1_HUMAN	1 (1)	31.36	8.03E-03	2.26
sp|Q9UBD9|CLCF1_HUMAN	1 (0)	31.17	---	---
sp|Q8NBJ4|GOLM1_HUMAN	1 (1)	31.15	0.67	1.16
sp|Q9NU53|GINM1_HUMAN	1 (1)	31.04	0.05	1.32
sp|A8MU46|SMTL1_HUMAN	1 (0)	30.65	---	---
sp|Q9Y4F9|RIPR2_HUMAN	1 (1)	30.41	0.65	1.1

sp|Q6UXD5|SE6L2_HUMAN	1 (1)	30.3	0.23	1.28
sp|Q53TQ3|IN80D_HUMAN	1 (1)	29.96	0.23	1.21
sp|Q6T310|RSLBA_HUMAN	1 (0)	29.75	---	---
sp|Q13202|DUS8_HUMAN	1 (0)	29.53	---	---
sp|Q8NI17|IL31R_HUMAN	1 (0)	29.24	---	---
sp|Q9BXX0|EMIL2_HUMAN	1 (0)	29.21	---	---
sp|Q8N3K9|CMYA5_HUMAN	1 (1)	29.15	0.06	1.33
sp|P05166|PCCB_HUMAN	1 (1)	29.13	0.22	1.09
sp|Q9UIF9|BAZ2A_HUMAN	1 (0)	29.11	---	---
sp|Q2TBE0|C19L2_HUMAN	1 (0)	29.06	---	---
sp|Q15149|PLEC_HUMAN	1 (0)	29	---	---
sp|Q0VDD7|CS057_HUMAN	1 (1)	29	0.08	1.42
sp|P51812|KS6A3_HUMAN	1 (1)	28.99	0.84	1.01
sp|P20273|CD22_HUMAN	1 (1)	28.96	0.56	1.07
sp|Q9HBH5|RDH14_HUMAN	1 (1)	28.87	0.92	1.01
sp|Q99574|NEUS_HUMAN	1 (1)	28.78	0.63	1.09
sp|A6NML5|TM212_HUMAN	1 (1)	28.77	0.73	1.1
sp|Q92729|PTPRU_HUMAN	1 (1)	28.75	0.1	1.2
sp|Q9P275|UBP36_HUMAN	1 (0)	28.74	---	---
sp|Q8N8Q3|ENDOV_HUMAN	1 (1)	28.73	0.78	1.11
sp|O60279|SUSD5_HUMAN	1 (1)	28.62	0.12	1.3
sp|P54764|EPHA4_HUMAN	1 (1)	28.54	0.87	1.26

sp|Q9ULT0|TTC7A_HUMAN	1 (1)	28.47	0.17	1.27
sp|Q13634|CAD18_HUMAN	1 (0)	28.36	---	---
sp|Q86XE3|MICU3_HUMAN	1 (1)	28.36	0.18	1.14
sp|P01344|IGF2_HUMAN	1 (1)	28.22	1	1
sp|P21815|SIAL_HUMAN	1 (1)	28.19	0.63	1.09
sp|A0A075B6K4|LV310_HUMAN	1 (1)	28.16	0.02	1.65
sp|Q9H1J1|REN3A_HUMAN	1 (1)	27.97	0.87	1.02
sp|Q86Z14|KLOTB_HUMAN	1 (1)	27.93	0.55	1.05
sp|Q92797|SYMPK_HUMAN	1 (1)	27.87	0.54	1.08
sp|Q9UM47|NOTC3_HUMAN	1 (1)	27.83	0.29	1.18
sp|P24928|RPB1_HUMAN	1 (1)	27.81	0.8	1.07
sp|P0CG38|POTEI_HUMAN	1 (0)	27.77	---	---
sp|Q96PE2|ARHGH_HUMAN	1 (0)	27.2	---	---
sp|P14923|PLAK_HUMAN	1 (1)	27.17	0.3	1.22
sp|P51530|DNA2_HUMAN	1 (1)	27.1	0.28	1.2
sp|Q9Y6X8|ZHX2_HUMAN	1 (1)	26.96	0.19	1.16
sp|O43491|E41L2_HUMAN	1 (1)	26.91	0.87	1.22
sp|Q9BZ72|PITM2_HUMAN	1 (1)	26.89	0.48	1.06
sp|P22670|RFX1_HUMAN	1 (0)	26.87	---	---
sp|Q7L0J3|SV2A_HUMAN	1 (1)	26.79	0.14	1.16
sp|P51553|IDH3G_HUMAN	1 (0)	26.73	---	---

sp|Q659A1|ICE2_HUMAN	1 (0)	26.68	---	---
sp|P11532|DMD_HUMAN	1 (1)	26.61	0.63	1.03
sp|O43314|VIP2_HUMAN	1 (1)	26.56	0.2	1.51
sp|Q6NXT6|TAPT1_HUMAN	1 (0)	26.52	---	---
sp|P09603|CSF1_HUMAN	1 (1)	26.43	0.81	1.02
sp|Q9Y5W7|SNX14_HUMAN	1 (1)	26.41	0.85	1.03
sp|Q8IWU2|LMTK2_HUMAN	1 (1)	26.15	0.89	1.01
sp|A6NLP5|TTC36_HUMAN	1 (1)	26.05	0.08	1.17
sp|O76041|NEBL_HUMAN	1 (1)	26.03	0.26	1.23
sp|P17038|ZNF43_HUMAN	1 (1)	25.97	0.33	1.18
sp|Q14BN4|SLMAP_HUMAN	1 (1)	25.85	0.32	1.23
sp|Q9H583|HEAT1_HUMAN	1 (1)	25.74	0.4	1.04
sp|Q12904|AIMP1_HUMAN	1 (1)	25.58	0.07	1.21
sp|Q96PQ0|SORC2_HUMAN	1 (1)	25.52	0.42	1.07
sp|Q8IVW6|ARI3B_HUMAN	1 (1)	25.47	0.01	1.49
sp|Q99708|CTIP_HUMAN	1 (1)	25.32	0.13	1.26
sp|P78559|MAP1A_HUMAN	1 (1)	25.23	0.8	1.06
sp|Q6ZQQ6|WDR87_HUMAN	1 (1)	24.96	0.26	1.27
sp|Q6P9G9|ZN449_HUMAN	1 (1)	24.91	0.21	1.47
sp|Q9BXK5|B2L13_HUMAN	1 (1)	24.9	0.29	1.14
sp|Q9NRJ7|PCDBG_HUMAN	1 (1)	24.86	0.09	1.42

sp|Q8NGR9|OR1N2_HUMAN	1 (1)	24.79	0.82	1.13
sp|P0C7N4|T191B_HUMAN	1 (1)	24.74	0.56	5.38
sp|Q9Y5K6|CD2AP_HUMAN	1 (1)	24.71	0.45	1.17
sp|P16298|PP2BB_HUMAN	1 (1)	24.69	0.25	1.41
sp|Q14397|GCKR_HUMAN	1 (1)	24.44	0.14	1.43
sp|Q86YP4|P66A_HUMAN	1 (1)	24.41	0.55	1.14
sp|Q08379|GOGA2_HUMAN	1 (1)	24.33	0.68	1.02
sp|Q7RTS7|K2C74_HUMAN	1 (1)	24.28	0.36	1.17
sp|P15408|FOSL2_HUMAN	1 (1)	24.26	0.95	1
sp|O15541|R113A_HUMAN	1 (0)	24.09	---	---
sp|Q9P281|BAHC1_HUMAN	1 (1)	24.06	0.8	1.02
sp|Q8IVL0|NAV3_HUMAN	1 (1)	24.04	2.93E-04	1.96
sp|Q9UJ99|CAD22_HUMAN	1 (1)	23.98	0.12	1.22
sp|P46108|CRK_HUMAN	1 (1)	23.86	0.2	1.34
sp|Q96JM4|LRIQ1_HUMAN	1 (1)	23.76	0.55	1.06
sp|Q9NS61|KCIP2_HUMAN	1 (1)	23.75	0.61	1.18
sp|Q8N9P6|CI163_HUMAN	1 (0)	23.74	---	---
sp|Q5YKI7|GGNB1_HUMAN	1 (1)	23.71	0.47	1.07
sp|Q03001|DYST_HUMAN	1 (0)	23.63	---	---
sp|P0C7V8|DC8L2_HUMAN	1 (1)	23.61	0.15	1.13
sp|P23975|SC6A2_HUMAN	1 (1)	23.61	0.07	1.37
sp|Q9Y3D8|KAD6_HUMAN	1 (1)	23.6	0.06	1.3

sp|Q9C0F3|ZN436_HUMAN	1 (1)	23.58	0.34	1.3
sp|P04090|REL2_HUMAN	1 (1)	23.54	0.03	1.55
sp|Q99418|CYH2_HUMAN	1 (0)	23.42	---	---
sp|Q9BXB5|OSB10_HUMAN	1 (1)	23.35	0.51	1.08
sp|Q14697|GANAB_HUMAN	1 (1)	23.31	0.11	1.57
sp|Q86YQ8|CPNE8_HUMAN	1 (1)	23.28	0.14	1.56
sp|Q8WY36|BBX_HUMAN	1 (1)	23.12	0.89	1.15
sp|Q9HCS7|SYF1_HUMAN	1 (0)	23.11	---	---
sp|A0A0A6YYD4|TVB13_HUMAN	1 (1)	23.06	0.04	1.5
sp|P0DPD6|ECE2_HUMAN	1 (1)	22.98	0.04	1.22
sp|O60307|MAST3_HUMAN	1 (1)	22.91	0.48	1.11
sp|Q13094|LCP2_HUMAN	1 (1)	22.83	3.31E-04	1.53
sp|Q8NDZ4|DIA1_HUMAN	1 (0)	22.78	---	---
sp|Q9UL51|HCN2_HUMAN	1 (1)	22.78	0.33	1.5
sp|Q9BXU1|STK31_HUMAN	1 (1)	22.71	0.97	1.02
sp|O75157|T22D2_HUMAN	1 (1)	22.69	0.13	1.16
sp|Q16650|TBR1_HUMAN	1 (0)	22.52	---	---
sp|Q9HB19|PKHA2_HUMAN	1 (1)	22.37	0.87	1.12
sp|Q7Z4L9|PPR42_HUMAN	1 (0)	22.25	---	---
sp|Q9BWD1|THIC_HUMAN	1 (1)	22.22	0.12	1.24
sp|Q9Y5K1|SPO11_HUMAN	1 (1)	22.12	0.49	1.31

sp|O43293|DAPK3_HUMAN	1 (1)	22	0.04	1.65
sp|P54252|ATX3_HUMAN	1 (1)	21.99	0.44	1.04
sp|P35555|FBN1_HUMAN	1 (1)	21.98	0.98	1.01
sp|Q15811|ITSN1_HUMAN	1 (1)	21.93	0.47	1.08
sp|Q7Z2T5|TRM1L_HUMAN	1 (0)	21.83	---	---
sp|Q6IE81|JADE1_HUMAN	1 (1)	21.81	0.43	1.1
sp|O75128|COBL_HUMAN	1 (1)	21.76	0.05	1.6
sp|Q9Y4C4|MFHA1_HUMAN	1 (1)	21.74	0.49	1.08
sp|Q14562|DHX8_HUMAN	1 (1)	21.71	0.27	1.33
sp|Q5TGI0|FAXC_HUMAN	1 (0)	21.68	---	---
sp|P60174|TPIS_HUMAN	1 (1)	21.64	0.01	1.96
sp|P22304|IDS_HUMAN	1 (1)	21.56	0.54	1.03
sp|Q5S007|LRRK2_HUMAN	1 (1)	21.52	0.35	1.14
sp|Q9NQV8|PRDM8_HUMAN	1 (1)	21.49	0.03	1.28
sp|Q8N2Q7|NLGN1_HUMAN	1 (1)	21.48	0.12	1.14
sp|Q5TC63|GRTP1_HUMAN	1 (1)	21.39	0.87	1.05
sp|O00370|LORF2_HUMAN	1 (1)	21.05	1	1.02
sp|Q9UKK3|PARP4_HUMAN	1 (0)	21.05	---	---
sp|P52630|STAT2_HUMAN	1 (1)	20.95	0.79	1.08
sp|P20701|ITAL_HUMAN	1 (1)	20.86	0.48	1.17
sp|Q86Y22|CONA1_HUMAN	1 (1)	20.85	0.06	1.39
sp|Q6ZMI3|GLDN_HUMAN	1 (1)	20.83	1	1

sp|Q9BZQ8|NIBAN_HUMAN	1 (1)	20.83	0.23	1.14
sp|Q8TBN0|R3GEF_HUMAN	1 (1)	20.61	0.03	1.34
sp|O14975|S27A2_HUMAN	1 (0)	20.57	---	---
sp|P10828|THB_HUMAN	1 (1)	20.46	0.86	1.01
sp|Q9C0G6|DYH6_HUMAN	1 (1)	20.39	0.49	1.14
sp|Q8WY54|PPM1E_HUMAN	1 (1)	20.37	0.68	1.02
sp|Q6Y7W6|GGYF2_HUMAN	1 (1)	20.35	0.24	1.15
sp|P51608|MECP2_HUMAN	1 (1)	20.23	0.15	1.2
sp|P08237|PFKAM_HUMAN	1 (1)	20.2	0.11	1.83
sp|Q13428|TCOF_HUMAN	1 (1)	20.18	8.59E-03	1.34
sp|Q9NZJ4|SACS_HUMAN	1 (1)	20.17	0.15	1.21
sp|Q969Y0|NXPE3_HUMAN	1 (1)	20.05	0.05	1.67
sp|Q9Y5W8|SNX13_HUMAN	1 (1)	20.03	0.2	1.21
sp|Q6ZT21|TMPPE_HUMAN	1 (1)	20.02	0.86	1.25

Description	Average Normalised	
	Ctrl	MDD
Serum albumin OS=Homo sapiens OX=9606 GN=ALB PE=1 SV=2	1.31E+08	1.12E+08
Complement C3 OS=Homo sapiens OX=9606 GN=C3 PE=1 SV=2	1.16E+06	1.21E+06
Serotransferrin OS=Homo sapiens OX=9606 GN=TF PE=1 SV=3	7.90E+06	7.82E+06
Complement C4-B OS=Homo sapiens OX=9606 GN=C4B PE=1 SV=2	5.19E+05	3.47E+05
Alpha-1-antitrypsin OS=Homo sapiens OX=9606 GN=SERPINA1 PE=1 SV=3	3.68E+06	6.06E+06
Alpha-2-macroglobulin OS=Homo sapiens OX=9606 GN=A2M PE=1 SV=3	3.37E+05	4.17E+05
Fibronectin OS=Homo sapiens OX=9606 GN=FN1 PE=1 SV=4	2.35E+05	2.62E+05
Clusterin OS=Homo sapiens OX=9606 GN=CLU PE=1 SV=1	3.15E+06	3.94E+06
Vitamin D-binding protein OS=Homo sapiens OX=9606 GN=GC PE=1 SV=1	1.88E+06	1.58E+06
Apolipoprotein E OS=Homo sapiens OX=9606 GN=APOE PE=1 SV=1	3.92E+06	4.38E+06
Secretogranin-1 OS=Homo sapiens OX=9606 GN=CHGB PE=1 SV=2	1.09E+06	1.27E+06
Neural cell adhesion molecule L1-like protein OS=Homo sapiens OX=9606 GN=CHL1 PE=1 SV=4	1.72E+05	1.96E+05
Apolipoprotein A-I OS=Homo sapiens OX=9606 GN=APOA1 PE=1 SV=1	2.16E+06	2.04E+06
Gelsolin OS=Homo sapiens OX=9606 GN=GSN PE=1 SV=1	7.22E+05	7.14E+05
Haptoglobin OS=Homo sapiens OX=9606 GN=HP PE=1 SV=1	8.86E+05	1.31E+06
SPARC-like protein 1 OS=Homo sapiens OX=9606 GN=SPARCL1 PE=1 SV=2	2.24E+05	2.44E+05
Transthyretin OS=Homo sapiens OX=9606 GN=TTR PE=1 SV=1	1.71E+07	1.41E+07
Ceruloplasmin OS=Homo sapiens OX=9606 GN=CP PE=1 SV=1	3.38E+05	4.01E+05
Antithrombin-III OS=Homo sapiens OX=9606 GN=SERPINC1 PE=1 SV=1	4.13E+05	3.22E+05
Amyloid-beta A4 protein OS=Homo sapiens OX=9606 GN=APP PE=1 SV=3	4.08E+05	4.13E+05
Immunoglobulin gamma-1 heavy chain OS=Homo sapiens OX=9606 PE=1 SV=2	7.67E+06	8.19E+06

Ectonucleotide pyrophosphatase/phosphodiesterase family member 2 OS=Homo sapiens OX=9606 GN=ENPP2 PE=1 SV=3	4.74E+05	4.78E+05
Hemopexin OS=Homo sapiens OX=9606 GN=HPX PE=1 SV=2	3.04E+06	2.53E+06
Apolipoprotein A-IV OS=Homo sapiens OX=9606 GN=APOA4 PE=1 SV=3	1.31E+05	1.16E+05
Neuronal cell adhesion molecule OS=Homo sapiens OX=9606 GN=NRCAM PE=1 SV=3	2.15E+05	2.68E+05
Angiotensinogen OS=Homo sapiens OX=9606 GN=AGT PE=1 SV=1	5.77E+05	7.66E+05
Immunoglobulin heavy constant gamma 3 OS=Homo sapiens OX=9606 GN=IGHG3 PE=1 SV=2	4.84E+05	4.16E+05
Fibulin-1 OS=Homo sapiens OX=9606 GN=FBLN1 PE=1 SV=4	1.01E+06	1.11E+06
Prostaglandin-H2 D-isomerase OS=Homo sapiens OX=9606 GN=PTGDS PE=1 SV=1	2.02E+07	2.03E+07
Pigment epithelium-derived factor OS=Homo sapiens OX=9606 GN=SERPINF1 PE=1 SV=4	8.65E+05	9.03E+05
Hemoglobin subunit beta OS=Homo sapiens OX=9606 GN=HBB PE=1 SV=2	7.33E+05	1.13E+06
Dickkopf-related protein 3 OS=Homo sapiens OX=9606 GN=DKK3 PE=1 SV=2	1.12E+06	1.29E+06
Chromogranin-A OS=Homo sapiens OX=9606 GN=CHGA PE=1 SV=7	1.22E+06	1.66E+06
Complement factor H OS=Homo sapiens OX=9606 GN=CFH PE=1 SV=4	1.76E+05	1.62E+05
Calsyntenin-1 OS=Homo sapiens OX=9606 GN=CLSTN1 PE=1 SV=1	1.17E+05	1.31E+05
Kallikrein-6 OS=Homo sapiens OX=9606 GN=KLK6 PE=1 SV=1	6.29E+05	7.06E+05
Neurosecretory protein VGF OS=Homo sapiens OX=9606 GN=VGF PE=1 SV=2	5.54E+05	6.90E+05
Amyloid-like protein 1 OS=Homo sapiens OX=9606 GN=APLP1 PE=1 SV=3	2.89E+05	3.12E+05
Alpha-1-acid glycoprotein 1 OS=Homo sapiens OX=9606 GN=ORM1 PE=1 SV=1	2.16E+06	2.21E+06
Alpha-1-acid glycoprotein 2 OS=Homo sapiens OX=9606 GN=ORM2 PE=1 SV=2	6.56E+05	5.14E+05
Immunoglobulin heavy constant alpha 1 OS=Homo sapiens OX=9606 GN=IGHA1 PE=1 SV=2	7.12E+05	9.16E+05
Secretogranin-3 OS=Homo sapiens OX=9606 GN=SCG3 PE=1 SV=3	6.07E+05	6.65E+05
Beta-Ala-His dipeptidase OS=Homo sapiens OX=9606 GN=CNDP1 PE=1 SV=4	2.89E+05	3.62E+05

Prothrombin OS=Homo sapiens OX=9606 GN=F2 PE=1 SV=2	3.01E+05	3.14E+05
Immunoglobulin heavy constant gamma 2 OS=Homo sapiens OX=9606 GN=IGHG2 PE=1 SV=2	2.28E+06	1.75E+06
Hemoglobin subunit alpha OS=Homo sapiens OX=9606 GN=HBA1 PE=1 SV=2	2.59E+05	4.15E+05
Complement factor B OS=Homo sapiens OX=9606 GN=CFB PE=1 SV=2	3.28E+05	3.05E+05
Immunoglobulin kappa light chain OS=Homo sapiens OX=9606 PE=1 SV=1	6.97E+05	8.44E+05
EGF-containing fibulin-like extracellular matrix protein 1 OS=Homo sapiens OX=9606 GN=EFEMP1 PE=1 SV=2	7.28E+05	8.41E+05
Neural cell adhesion molecule 1 OS=Homo sapiens OX=9606 GN=NCAM1 PE=1 SV=3	1.46E+05	1.72E+05
Cystatin-C OS=Homo sapiens OX=9606 GN=CST3 PE=1 SV=1	8.96E+06	1.09E+07
Alpha-2-HS-glycoprotein OS=Homo sapiens OX=9606 GN=AHSG PE=1 SV=1	5.65E+05	4.07E+05
Immunoglobulin kappa constant OS=Homo sapiens OX=9606 GN=IGKC PE=1 SV=2	7.65E+06	7.86E+06
Plasminogen OS=Homo sapiens OX=9606 GN=PLG PE=1 SV=2	2.01E+05	1.67E+05
Contactin-1 OS=Homo sapiens OX=9606 GN=CNTN1 PE=1 SV=1	9.23E+04	9.60E+04
Insulin-like growth factor-binding protein 7 OS=Homo sapiens OX=9606 GN=IGFBP7 PE=1 SV=1	2.40E+05	2.12E+05
Histidine-rich glycoprotein OS=Homo sapiens OX=9606 GN=HRG PE=1 SV=1	1.21E+05	1.24E+05
Protein FAM3C OS=Homo sapiens OX=9606 GN=FAM3C PE=1 SV=1	2.96E+05	3.13E+05
Keratin, type I cytoskeletal 10 OS=Homo sapiens OX=9606 GN=KRT10 PE=1 SV=6	5.28E+04	1.01E+05
Immunoglobulin lambda constant 2 OS=Homo sapiens OX=9606 GN=IGLC2 PE=1 SV=1	3.01E+06	2.92E+06
Immunoglobulin lambda-like polypeptide 5 OS=Homo sapiens OX=9606 GN=IGLL5 PE=2 SV=2	1.06E+06	1.10E+06
Kininogen-1 OS=Homo sapiens OX=9606 GN=KNG1 PE=1 SV=2	2.42E+05	2.10E+05
Plasma protease C1 inhibitor OS=Homo sapiens OX=9606 GN=SERPING1 PE=1 SV=2	3.15E+05	3.13E+05
Immunoglobulin heavy constant gamma 4 OS=Homo sapiens OX=9606 GN=IGHG4 PE=1 SV=1	4.71E+05	4.44E+05
Fibrinogen gamma chain OS=Homo sapiens OX=9606 GN=FGG PE=1 SV=3	9.47E+04	1.56E+05

Alpha-1-antichymotrypsin OS=Homo sapiens OX=9606 GN=SERPINA3 PE=1 SV=2	5.89E+05	6.67E+05
Protein kinase C-binding protein NELL2 OS=Homo sapiens OX=9606 GN=NELL2 PE=1 SV=1	8.66E+04	1.15E+05
Mimecan OS=Homo sapiens OX=9606 GN=OGN PE=1 SV=1	7.01E+04	6.91E+04
Keratin, type II cytoskeletal 1 OS=Homo sapiens OX=9606 GN=KRT1 PE=1 SV=6	3.33E+04	7.03E+04
Galectin-3-binding protein OS=Homo sapiens OX=9606 GN=LGALS3BP PE=1 SV=1	1.56E+05	1.58E+05
ProSAAS OS=Homo sapiens OX=9606 GN=PCSK1N PE=1 SV=1	5.83E+05	5.50E+05
Inter-alpha-trypsin inhibitor heavy chain H4 OS=Homo sapiens OX=9606 GN=ITIH4 PE=1 SV=4	2.76E+04	2.70E+04
Complement component C7 OS=Homo sapiens OX=9606 GN=C7 PE=1 SV=2	2.92E+05	2.91E+05
Alpha-1B-glycoprotein OS=Homo sapiens OX=9606 GN=A1BG PE=1 SV=4	1.41E+05	1.24E+05
Fibrinogen beta chain OS=Homo sapiens OX=9606 GN=FGB PE=1 SV=2	1.36E+05	2.13E+05
Insulin-like growth factor-binding protein 2 OS=Homo sapiens OX=9606 GN=IGFBP2 PE=1 SV=2	2.06E+05	2.01E+05
Cathepsin D OS=Homo sapiens OX=9606 GN=CTSD PE=1 SV=1	3.29E+05	2.76E+05
Beta-1,4-glucuronyltransferase 1 OS=Homo sapiens OX=9606 GN=B4GAT1 PE=1 SV=1	1.69E+05	2.09E+05
Keratin, type I cytoskeletal 9 OS=Homo sapiens OX=9606 GN=KRT9 PE=1 SV=3	9.50E+04	1.44E+05
Neuronal pentraxin-1 OS=Homo sapiens OX=9606 GN=NPTX1 PE=2 SV=2	8.35E+04	8.82E+04
Osteopontin OS=Homo sapiens OX=9606 GN=SPP1 PE=1 SV=1	5.03E+05	5.69E+05
Fibrinogen alpha chain OS=Homo sapiens OX=9606 GN=FGA PE=1 SV=2	3.62E+04	4.98E+04
Apolipoprotein A-II OS=Homo sapiens OX=9606 GN=APOA2 PE=1 SV=1	8.71E+05	6.68E+05
Monocyte differentiation antigen CD14 OS=Homo sapiens OX=9606 GN=CD14 PE=1 SV=2	7.14E+04	8.86E+04
Afamin OS=Homo sapiens OX=9606 GN=AFM PE=1 SV=1	8.85E+04	8.20E+04
Beta-2-glycoprotein 1 OS=Homo sapiens OX=9606 GN=APOH PE=1 SV=3	6.47E+05	4.33E+05
Prosaposin OS=Homo sapiens OX=9606 GN=PSAP PE=1 SV=2	1.58E+05	1.86E+05

Neuronal pentraxin receptor OS=Homo sapiens OX=9606 GN=NPTXR PE=3 SV=2	2.47E+05	2.57E+05
Secretogranin-2 OS=Homo sapiens OX=9606 GN=SCG2 PE=1 SV=2	2.25E+05	2.71E+05
Immunoglobulin alpha-2 heavy chain OS=Homo sapiens OX=9606 PE=1 SV=2	9241.13	9398.88
Inter-alpha-trypsin inhibitor heavy chain H2 OS=Homo sapiens OX=9606 GN=ITIH2 PE=1 SV=2	4.29E+04	4.10E+04
Superoxide dismutase [Cu-Zn] OS=Homo sapiens OX=9606 GN=SOD1 PE=1 SV=2	3.83E+05	4.29E+05
Cadherin-13 OS=Homo sapiens OX=9606 GN=CDH13 PE=1 SV=1	3.61E+04	4.78E+04
Beta-2-microglobulin OS=Homo sapiens OX=9606 GN=B2M PE=1 SV=1	1.16E+06	1.30E+06
Leucine-rich alpha-2-glycoprotein OS=Homo sapiens OX=9606 GN=LRG1 PE=1 SV=2	3.26E+04	4.11E+04
Apolipoprotein D OS=Homo sapiens OX=9606 GN=APOD PE=1 SV=1	1.83E+06	1.91E+06
Complement factor I OS=Homo sapiens OX=9606 GN=CFI PE=1 SV=2	5.48E+04	5.40E+04
Complement C1s subcomponent OS=Homo sapiens OX=9606 GN=C1S PE=1 SV=1	1.42E+05	1.60E+05
Ganglioside GM2 activator OS=Homo sapiens OX=9606 GN=GM2A PE=1 SV=4	5.15E+04	6.22E+04
Heparin cofactor 2 OS=Homo sapiens OX=9606 GN=SERPIND1 PE=1 SV=3	6.38E+04	6.20E+04
Tetranectin OS=Homo sapiens OX=9606 GN=CLEC3B PE=1 SV=3	1.35E+05	1.12E+05
IgGFc-binding protein OS=Homo sapiens OX=9606 GN=FCGBP PE=1 SV=3	3.78E+04	2.57E+04
Immunoglobulin heavy constant alpha 2 OS=Homo sapiens OX=9606 GN=IGHA2 PE=1 SV=4	---	---
Chitinase-3-like protein 1 OS=Homo sapiens OX=9606 GN=CHI3L1 PE=1 SV=2	4.69E+04	6.47E+04
Collagen alpha-1(VI) chain OS=Homo sapiens OX=9606 GN=COL6A1 PE=1 SV=3	4.84E+04	5.27E+04
Keratin, type II cytoskeletal 2 epidermal OS=Homo sapiens OX=9606 GN=KRT2 PE=1 SV=2	1.82E+05	2.37E+05
NPC intracellular cholesterol transporter 2 OS=Homo sapiens OX=9606 GN=NPC2 PE=1 SV=1	2.29E+05	2.40E+05
Alpha-2-antiplasmin OS=Homo sapiens OX=9606 GN=SERPINF2 PE=1 SV=3	6.43E+04	5.01E+04
Ribonuclease pancreatic OS=Homo sapiens OX=9606 GN=RNASE1 PE=1 SV=4	1.04E+05	1.06E+05

Extracellular superoxide dismutase [Cu-Zn] OS=Homo sapiens OX=9606 GN=SOD3 PE=1 SV=2	4.54E+04	4.52E+04
Complement component C9 OS=Homo sapiens OX=9606 GN=C9 PE=1 SV=2	9.61E+04	1.00E+05
Phosphatidylethanolamine-binding protein 1 OS=Homo sapiens OX=9606 GN=PEBP1 PE=1 SV=3	1.80E+05	2.04E+05
SPARC OS=Homo sapiens OX=9606 GN=SPARC PE=1 SV=1	3.63E+04	3.71E+04
Immunoglobulin mu heavy chain OS=Homo sapiens OX=9606 PE=1 SV=2	2.23E+04	4.21E+04
Tyrosine-protein phosphatase non-receptor type substrate 1 OS=Homo sapiens OX=9606 GN=SIRPA PE=1 SV=2	4.78E+04	5.96E+04
Contactin-2 OS=Homo sapiens OX=9606 GN=CNTN2 PE=1 SV=1	1.88E+04	1.86E+04
Dystroglycan OS=Homo sapiens OX=9606 GN=DAG1 PE=1 SV=2	9.36E+04	1.15E+05
Insulin-like growth factor-binding protein 6 OS=Homo sapiens OX=9606 GN=IGFBP6 PE=1 SV=1	2.75E+05	2.81E+05
Proenkephalin-A OS=Homo sapiens OX=9606 GN=PENK PE=1 SV=1	2.65E+05	2.66E+05
Brevican core protein OS=Homo sapiens OX=9606 GN=BCAN PE=1 SV=2	1.86E+04	2.41E+04
Neurotrimin OS=Homo sapiens OX=9606 GN=NTM PE=1 SV=1	1.35E+05	1.60E+05
Extracellular matrix protein 1 OS=Homo sapiens OX=9606 GN=ECM1 PE=1 SV=2	7.22E+04	9.22E+04
Pyruvate kinase PKM OS=Homo sapiens OX=9606 GN=PKM PE=1 SV=4	3.61E+04	3.84E+04
Vitronectin OS=Homo sapiens OX=9606 GN=VTN PE=1 SV=1	1.49E+05	1.23E+05
Complement C1q subcomponent subunit C OS=Homo sapiens OX=9606 GN=C1QC PE=1 SV=3	9.48E+04	1.20E+05
Amyloid-like protein 2 OS=Homo sapiens OX=9606 GN=APLP2 PE=1 SV=2	1.90E+04	2.06E+04
Peptidyl-glycine alpha-amidating monooxygenase OS=Homo sapiens OX=9606 GN=PAM PE=1 SV=2	1.94E+04	2.08E+04
Thy-1 membrane glycoprotein OS=Homo sapiens OX=9606 GN=THY1 PE=1 SV=2	5.99E+04	7.75E+04
Cartilage acidic protein 1 OS=Homo sapiens OX=9606 GN=CRTAC1 PE=1 SV=2	7.73E+04	8.03E+04
Immunoglobulin lambda variable 3-9 OS=Homo sapiens OX=9606 GN=IGLV3-9 PE=3 SV=1	1.12E+05	1.26E+05
Neuroendocrine protein 7B2 OS=Homo sapiens OX=9606 GN=SCG5 PE=1 SV=2	1.85E+05	2.12E+05

Cadherin-2 OS=Homo sapiens OX=9606 GN=CDH2 PE=1 SV=4	8.37E+04	1.01E+05
Phosphoinositide-3-kinase-interacting protein 1 OS=Homo sapiens OX=9606 GN=PIK3IP1 PE=1 SV=2	1.32E+05	1.46E+05
Complement C1q subcomponent subunit B OS=Homo sapiens OX=9606 GN=C1QB PE=1 SV=3	5.08E+04	6.26E+04
Neurocan core protein OS=Homo sapiens OX=9606 GN=NCAN PE=1 SV=3	3.50E+04	3.58E+04
Neuroblastoma suppressor of tumorigenicity 1 OS=Homo sapiens OX=9606 GN=NBL1 PE=1 SV=2	1.93E+04	2.21E+04
Protein AMBP OS=Homo sapiens OX=9606 GN=AMBP PE=1 SV=1	4.14E+04	4.23E+04
Major prion protein OS=Homo sapiens OX=9606 GN=PRNP PE=1 SV=1	2.88E+04	2.80E+04
Scrapie-responsive protein 1 OS=Homo sapiens OX=9606 GN=SCRG1 PE=1 SV=1	2.10E+04	2.18E+04
Complement C1r subcomponent OS=Homo sapiens OX=9606 GN=C1R PE=1 SV=2	7.48E+04	7.49E+04
Limbic system-associated membrane protein OS=Homo sapiens OX=9606 GN=LSAMP PE=1 SV=2	1.14E+05	1.39E+05
Ubiquitin-40S ribosomal protein S27a OS=Homo sapiens OX=9606 GN=RPS27A PE=1 SV=2	3.31E+05	3.21E+05
Neural cell adhesion molecule 2 OS=Homo sapiens OX=9606 GN=NCAM2 PE=1 SV=2	2.93E+04	3.68E+04
N-acetylmuramoyl-L-alanine amidase OS=Homo sapiens OX=9606 GN=PGLYRP2 PE=1 SV=1	3.19E+04	2.52E+04
CD59 glycoprotein OS=Homo sapiens OX=9606 GN=CD59 PE=1 SV=1	1.31E+05	1.46E+05
Retinol-binding protein 4 OS=Homo sapiens OX=9606 GN=RBP4 PE=1 SV=3	8.31E+04	6.95E+04
Corticosteroid-binding globulin OS=Homo sapiens OX=9606 GN=SERPINA6 PE=1 SV=1	1.13E+04	1.25E+04
Complement C2 OS=Homo sapiens OX=9606 GN=C2 PE=1 SV=2	8897.69	9280.09
Immunoglobulin heavy variable 3-30-5 OS=Homo sapiens OX=9606 GN=IGHV3-30-5 PE=3 SV=1	5.29E+04	5.05E+04
Protein CutA OS=Homo sapiens OX=9606 GN=CUTA PE=1 SV=2	5.02E+04	5.51E+04
Transforming growth factor-beta-induced protein ig-h3 OS=Homo sapiens OX=9606 GN=TGFBI PE=1 SV=1	8014.92	8346.3
Metalloproteinase inhibitor 2 OS=Homo sapiens OX=9606 GN=TIMP2 PE=1 SV=2	1.50E+05	1.77E+05
Voltage-dependent calcium channel subunit alpha-2/delta-1 OS=Homo sapiens OX=9606 GN=CACNA2D1 PE=1 SV=3	2.80E+04	3.23E+04

Carboxypeptidase E OS=Homo sapiens OX=9606 GN=CPE PE=1 SV=1	2.76E+04	2.91E+04
Semaphorin-7A OS=Homo sapiens OX=9606 GN=SEMA7A PE=1 SV=1	2.75E+04	3.32E+04
V-type proton ATPase subunit S1 OS=Homo sapiens OX=9606 GN=ATP6AP1 PE=1 SV=2	4.65E+04	5.18E+04
CD99 antigen-like protein 2 OS=Homo sapiens OX=9606 GN=CD99L2 PE=1 SV=1	1.53E+04	1.91E+04
Extracellular sulfatase Sulf-2 OS=Homo sapiens OX=9606 GN=SULF2 PE=1 SV=1	1.67E+04	1.84E+04
Cell adhesion molecule 3 OS=Homo sapiens OX=9606 GN=CADM3 PE=1 SV=1	5.99E+04	5.98E+04
Cell adhesion molecule 1 OS=Homo sapiens OX=9606 GN=CADM1 PE=1 SV=2	8.22E+04	8.86E+04
Neural proliferation differentiation and control protein 1 OS=Homo sapiens OX=9606 GN=NPDC1 PE=1 SV=2	2.79E+04	3.09E+04
Chondroitin sulfate proteoglycan 5 OS=Homo sapiens OX=9606 GN=CSPG5 PE=1 SV=3	7.27E+04	7.89E+04
Immunoglobulin heavy variable 3-74 OS=Homo sapiens OX=9606 GN=IGHV3-74 PE=3 SV=1	8505.82	9503.46
Immunoglobulin kappa variable 3-20 OS=Homo sapiens OX=9606 GN=IGKV3-20 PE=1 SV=2	2.89E+04	3.61E+04
Procollagen C-endopeptidase enhancer 1 OS=Homo sapiens OX=9606 GN=PCOLCE PE=1 SV=2	7.62E+04	6.60E+04
Reelin OS=Homo sapiens OX=9606 GN=RELN PE=1 SV=3	1.27E+04	1.14E+04
Neurofascin OS=Homo sapiens OX=9606 GN=NFASC PE=1 SV=4	1.25E+04	1.33E+04
Apolipoprotein C-III OS=Homo sapiens OX=9606 GN=APOC3 PE=1 SV=1	2.65E+05	2.73E+05
V-set and transmembrane domain-containing protein 2A OS=Homo sapiens OX=9606 GN=VSTM2A PE=1 SV=3	1.58E+04	2.02E+04
Receptor-type tyrosine-protein phosphatase zeta OS=Homo sapiens OX=9606 GN=PTPRZ1 PE=1 SV=4	1.76E+04	2.19E+04
Cell surface glycoprotein MUC18 OS=Homo sapiens OX=9606 GN=MCAM PE=1 SV=2	2.23E+04	2.71E+04
Complement C1q subcomponent subunit A OS=Homo sapiens OX=9606 GN=C1QA PE=1 SV=2	2.52E+04	2.78E+04
Immunoglobulin lambda variable 3-19 OS=Homo sapiens OX=9606 GN=IGLV3-19 PE=1 SV=2	4221.38	4465.23
Immunoglobulin kappa variable 3D-7 OS=Homo sapiens OX=9606 GN=IGKV3D-7 PE=3 SV=5	4.77E+04	4.33E+04

Immunoglobulin kappa variable 2D-29 OS=Homo sapiens OX=9606 GN=IGKV2D-29 PE=3 SV=1	817.78	1812.46
Receptor-type tyrosine-protein phosphatase N2 OS=Homo sapiens OX=9606 GN=PTPRN2 PE=1 SV=2	5.26E+04	6.20E+04
Macrophage colony-stimulating factor 1 receptor OS=Homo sapiens OX=9606 GN=CSF1R PE=1 SV=2	3.47E+04	4.15E+04
Immunoglobulin lambda variable 1-51 OS=Homo sapiens OX=9606 GN=IGLV1-51 PE=1 SV=2	6237.26	1.10E+04
FERM and PDZ domain-containing protein 1 OS=Homo sapiens OX=9606 GN=FRMPD1 PE=1 SV=1	3781.41	3605.86
Aspartate aminotransferase, cytoplasmic OS=Homo sapiens OX=9606 GN=GOT1 PE=1 SV=3	1.05E+04	1.13E+04
Glutamate receptor 4 OS=Homo sapiens OX=9606 GN=GRIA4 PE=1 SV=2	5485.14	1.04E+04
Neogenin OS=Homo sapiens OX=9606 GN=NEO1 PE=1 SV=2	7553.99	1.02E+04
Immunoglobulin lambda variable 1-47 OS=Homo sapiens OX=9606 GN=IGLV1-47 PE=1 SV=2	3.84E+04	3.90E+04
Neurexin-1-beta OS=Homo sapiens OX=9606 GN=NRXN1 PE=1 SV=3	5.22E+04	6.94E+04
Pro-neuropeptide Y OS=Homo sapiens OX=9606 GN=NPY PE=1 SV=1	1.02E+04	1.10E+04
Glyceraldehyde-3-phosphate dehydrogenase OS=Homo sapiens OX=9606 GN=GAPDH PE=1 SV=3	2.53E+04	2.89E+04
Somatostatin OS=Homo sapiens OX=9606 GN=SST PE=1 SV=1	1705.08	1200.7
Cell adhesion molecule 4 OS=Homo sapiens OX=9606 GN=CADM4 PE=1 SV=1	3242.81	4328.4
Neurexin-3-beta OS=Homo sapiens OX=9606 GN=NRXN3 PE=1 SV=4	1.37E+04	1.21E+04
SPATS2-like protein OS=Homo sapiens OX=9606 GN=SPATS2L PE=1 SV=2	4.79E+04	4.49E+04
Vitamin K-dependent protein S OS=Homo sapiens OX=9606 GN=PROS1 PE=1 SV=1	2.54E+04	2.48E+04
Receptor-type tyrosine-protein phosphatase S OS=Homo sapiens OX=9606 GN=PTPRS PE=1 SV=3	3.80E+04	4.66E+04
Collagen alpha-2(I) chain OS=Homo sapiens OX=9606 GN=COL1A2 PE=1 SV=7	3.31E+04	2.94E+04
Kallistatin OS=Homo sapiens OX=9606 GN=SERPINA4 PE=1 SV=3	6168	2980.18
Nucleobindin-1 OS=Homo sapiens OX=9606 GN=NUCB1 PE=1 SV=4	1.05E+04	1.01E+04
Biotinidase OS=Homo sapiens OX=9606 GN=BTD PE=1 SV=2	2.01E+04	2.11E+04

Protein YIPF3 OS=Homo sapiens OX=9606 GN=YIPF3 PE=1 SV=1	7.62E+04	6.85E+04
Peptidyl-prolyl cis-trans isomerase B OS=Homo sapiens OX=9606 GN=PPIB PE=1 SV=2	1.41E+04	1.36E+04
Phospholipid transfer protein OS=Homo sapiens OX=9606 GN=PLTP PE=1 SV=1	5.11E+04	5.97E+04
Thyroxine-binding globulin OS=Homo sapiens OX=9606 GN=SERPINA7 PE=1 SV=2	1.60E+04	1.68E+04
Lumican OS=Homo sapiens OX=9606 GN=LUM PE=1 SV=2	1373.81	2188.12
Immunoglobulin heavy variable 3-21 OS=Homo sapiens OX=9606 GN=IGHV3-21 PE=1 SV=1	---	---
Neuronal growth regulator 1 OS=Homo sapiens OX=9606 GN=NEGR1 PE=1 SV=3	7.49E+04	1.01E+05
Cocaine- and amphetamine-regulated transcript protein OS=Homo sapiens OX=9606 GN=CARTPT PE=1 SV=1	4671.11	6136.79
Fructose-bisphosphate aldolase A OS=Homo sapiens OX=9606 GN=ALDOA PE=1 SV=2	2.17E+04	2.66E+04
Tetratricopeptide repeat protein 37 OS=Homo sapiens OX=9606 GN=TTC37 PE=1 SV=1	---	---
CD44 antigen OS=Homo sapiens OX=9606 GN=CD44 PE=1 SV=3	1.56E+04	2.36E+04
Serum amyloid A-4 protein OS=Homo sapiens OX=9606 GN=SAA4 PE=1 SV=2	---	---
Calsyntenin-3 OS=Homo sapiens OX=9606 GN=CLSTN3 PE=1 SV=1	1.63E+04	1.07E+04
Retinoic acid receptor responder protein 2 OS=Homo sapiens OX=9606 GN=RARRES2 PE=1 SV=1	2.56E+04	2.79E+04
Lysosome-associated membrane glycoprotein 2 OS=Homo sapiens OX=9606 GN=LAMP2 PE=1 SV=2	1.60E+04	2.27E+04
Coiled-coil domain-containing protein 40 OS=Homo sapiens OX=9606 GN=CCDC40 PE=2 SV=2	1.93E+04	2.15E+04
Leucine-rich repeat-containing protein 4B OS=Homo sapiens OX=9606 GN=LRRC4B PE=2 SV=3	1.86E+04	2.01E+04
Exosome complex component RRP43 OS=Homo sapiens OX=9606 GN=EXOSC8 PE=1 SV=1	---	---
Opioid-binding protein/cell adhesion molecule OS=Homo sapiens OX=9606 GN=OPCML PE=1 SV=1	3.10E+04	3.75E+04
Transmembrane protein 198 OS=Homo sapiens OX=9606 GN=TMEM198 PE=1 SV=1	3.44E+05	3.06E+05
Tumor necrosis factor receptor superfamily member 21 OS=Homo sapiens OX=9606 GN=TNFRSF21 PE=1 SV=1	1.80E+04	2.19E+04

Glutaminyl-peptide cyclotransferase OS=Homo sapiens OX=9606 GN=QPCT PE=1 SV=1	3007.36	7047.94
Proteasome subunit alpha type-1 OS=Homo sapiens OX=9606 GN=PSMA1 PE=1 SV=1	---	---
Coagulation factor XII OS=Homo sapiens OX=9606 GN=F12 PE=1 SV=3	1739.48	1621.31
Cathepsin L1 OS=Homo sapiens OX=9606 GN=CTSL PE=1 SV=2	5.12E+04	5.11E+04
Apolipoprotein C-I OS=Homo sapiens OX=9606 GN=APOC1 PE=1 SV=1	4.48E+04	4.92E+04
Protein C3orf33 OS=Homo sapiens OX=9606 GN=C3orf33 PE=1 SV=2	3.94E+05	2.72E+05
Titin OS=Homo sapiens OX=9606 GN=TTN PE=1 SV=4	3.12E+04	2.73E+04
Immunoglobulin kappa variable 4-1 OS=Homo sapiens OX=9606 GN=IGKV4-1 PE=1 SV=1	3.02E+04	2.73E+04
Ecto-ADP-ribosyltransferase 4 OS=Homo sapiens OX=9606 GN=ART4 PE=2 SV=2	---	---
Complement component C6 OS=Homo sapiens OX=9606 GN=C6 PE=1 SV=3	3710.91	2186.87
Polyamine-modulated factor 1-binding protein 1 OS=Homo sapiens OX=9606 GN=PMFBP1 PE=2 SV=2	4.18E+05	4.71E+05
Disintegrin and metalloproteinase domain-containing protein 22 OS=Homo sapiens OX=9606 GN=ADAM22 PE=1 SV=1	2851.1	2390.61
Olfactory receptor 4B1 OS=Homo sapiens OX=9606 GN=OR4B1 PE=3 SV=1	7.39E+04	7.26E+04
GREB1-like protein OS=Homo sapiens OX=9606 GN=GREB1L PE=1 SV=2	3.39E+05	2.86E+05
Complement factor D OS=Homo sapiens OX=9606 GN=CFD PE=1 SV=5	1.47E+04	2.42E+04
Complement component C8 gamma chain OS=Homo sapiens OX=9606 GN=C8G PE=1 SV=3	8599.38	7438.28
Centrosomal protein of 290 kDa OS=Homo sapiens OX=9606 GN=CEP290 PE=1 SV=2	---	---
DNA replication factor Cdt1 OS=Homo sapiens OX=9606 GN=CDT1 PE=1 SV=3	---	---
Arfaptin-1 OS=Homo sapiens OX=9606 GN=ARFIP1 PE=1 SV=2	---	---
Uncharacterized protein C1orf189 OS=Homo sapiens OX=9606 GN=C1orf189 PE=1 SV=1	3.50E+04	2.26E+04
Cholecystokinin OS=Homo sapiens OX=9606 GN=CCK PE=1 SV=1	1.01E+04	1.07E+04
ATP-dependent RNA helicase DDX55 OS=Homo sapiens OX=9606 GN=DDX55 PE=1 SV=3	1.26E+05	1.12E+05

Inactive serine/threonine-protein kinase VRK3 OS=Homo sapiens OX=9606 GN=VRK3 PE=1 SV=2	3.15E+04	3.70E+04
Exostosin-like 2 OS=Homo sapiens OX=9606 GN=EXTL2 PE=1 SV=1	1.67E+04	1.79E+04
Collagen alpha-1(XVIII) chain OS=Homo sapiens OX=9606 GN=COL18A1 PE=1 SV=5	6613.74	6988.34
Centrosomal protein C10orf90 OS=Homo sapiens OX=9606 GN=C10orf90 PE=2 SV=2	---	---
MAP7 domain-containing protein 2 OS=Homo sapiens OX=9606 GN=MAP7D2 PE=1 SV=2	8.95E+04	8.50E+04
Protein ELYS OS=Homo sapiens OX=9606 GN=AHCTF1 PE=1 SV=3	9529.28	8523.51
Polynucleotide 5'-hydroxyl-kinase NOL9 OS=Homo sapiens OX=9606 GN=NOL9 PE=1 SV=1	1.20E+05	1.22E+05
Immunoglobulin kappa joining 1 OS=Homo sapiens OX=9606 GN=IGKJ1 PE=4 SV=2	2215.85	7872.5
Ankyrin repeat domain-containing protein 11 OS=Homo sapiens OX=9606 GN=ANKRD11 PE=1 SV=3	---	---
Neurexin-2 OS=Homo sapiens OX=9606 GN=NRXN2 PE=2 SV=1	1.48E+04	2.13E+04
ATP synthase subunit alpha, mitochondrial OS=Homo sapiens OX=9606 GN=ATP5F1A PE=1 SV=1	2.33E+04	1.66E+04
Calmodulin-1 OS=Homo sapiens OX=9606 GN=CALM1 PE=1 SV=1	7.08E+04	7.14E+04
Putative glycine N-acyltransferase-like protein 1B OS=Homo sapiens OX=9606 GN=GLYATL1B PE=3 SV=1	5.73E+04	6.23E+04
SEC23-interacting protein OS=Homo sapiens OX=9606 GN=SEC23IP PE=1 SV=1	1.51E+04	1.13E+04
Golgi resident protein GCP60 OS=Homo sapiens OX=9606 GN=ACBD3 PE=1 SV=4	2035.56	2634.44
Keratin, type II cuticular Hb1 OS=Homo sapiens OX=9606 GN=KRT81 PE=1 SV=3	4.35E+04	4.00E+04
Puromycin-sensitive aminopeptidase OS=Homo sapiens OX=9606 GN=NPEPPS PE=1 SV=2	---	---
14-3-3 protein theta OS=Homo sapiens OX=9606 GN=YWHAQ PE=1 SV=1	---	---
Serine palmitoyltransferase 3 OS=Homo sapiens OX=9606 GN=SPTLC3 PE=1 SV=3	718.28	839.82
Tubby-related protein 1 OS=Homo sapiens OX=9606 GN=TULP1 PE=1 SV=3	3.67E+05	2.44E+05
Zinc-alpha-2-glycoprotein OS=Homo sapiens OX=9606 GN=AZGP1 PE=1 SV=2	1.05E+04	7352.05
Intermediate filament family orphan 2 OS=Homo sapiens OX=9606 GN=IFFO2 PE=2 SV=3	1061.5	720.75

Mevalonate kinase OS=Homo sapiens OX=9606 GN=MVK PE=1 SV=1	7.02E+05	7.68E+05
PDZ domain-containing protein 9 OS=Homo sapiens OX=9606 GN=PDZD9 PE=2 SV=2	6.40E+04	6.20E+04
Patched domain-containing protein 3 OS=Homo sapiens OX=9606 GN=PTCHD3 PE=1 SV=3	1.86E+04	1.68E+04
Zinc finger and SCAN domain-containing protein 10 OS=Homo sapiens OX=9606 GN=ZSCAN10 PE=1 SV=1	---	---
Peroxisomal sarcosine oxidase OS=Homo sapiens OX=9606 GN=PIPOX PE=1 SV=2	3.10E+04	3.99E+04
Cyclic AMP-responsive element-binding protein 3-like protein 3 OS=Homo sapiens OX=9606 GN=CREB3L3 PE=1 SV=2	1599.89	3005.38
Immunoglobulin lambda-like polypeptide 1 OS=Homo sapiens OX=9606 GN=IGLL1 PE=1 SV=1	7.52E+05	6.95E+05
Oligodendrocyte-myelin glycoprotein OS=Homo sapiens OX=9606 GN=OMG PE=1 SV=2	9038.23	1.71E+04
Ly-6/neurotoxin-like protein 1 OS=Homo sapiens OX=9606 GN=LYNX1 PE=1 SV=1	7701.78	5663.9
Forkhead-associated domain-containing protein 1 OS=Homo sapiens OX=9606 GN=FHAD1 PE=2 SV=2	735.46	381.74
Coagulation factor V OS=Homo sapiens OX=9606 GN=F5 PE=1 SV=4	---	---
Protein diaphanous homolog 2 OS=Homo sapiens OX=9606 GN=DIAPH2 PE=1 SV=1	4.15E+04	4.10E+04
N-alpha-acetyltransferase 15, NatA auxiliary subunit OS=Homo sapiens OX=9606 GN=NAA15 PE=1 SV=1	---	---
Immunoglobulin heavy variable 3-15 OS=Homo sapiens OX=9606 GN=IGHV3-15 PE=3 SV=1	5.20E+04	4.63E+04
Tyrosine-protein kinase SYK OS=Homo sapiens OX=9606 GN=SYK PE=1 SV=1	---	---
Hedgehog-interacting protein OS=Homo sapiens OX=9606 GN=HHIP PE=1 SV=3	---	---
Serine/threonine-protein kinase/endoribonuclease IRE1 OS=Homo sapiens OX=9606 GN=ERN1 PE=1 SV=2	1.86E+04	4.20E+04
Cardiotrophin-like cytokine factor 1 OS=Homo sapiens OX=9606 GN=CLCF1 PE=1 SV=1	---	---
Golgi membrane protein 1 OS=Homo sapiens OX=9606 GN=GOLM1 PE=1 SV=1	1.43E+04	1.65E+04
Glycoprotein integral membrane protein 1 OS=Homo sapiens OX=9606 GN=GINM1 PE=2 SV=1	1.17E+05	8.92E+04
Smoothelin-like protein 1 OS=Homo sapiens OX=9606 GN=SMTNL1 PE=1 SV=1	---	---
Rho family-interacting cell polarization regulator 2 OS=Homo sapiens OX=9606 GN=RIPOR2 PE=1 SV=4	2.52E+04	2.29E+04

Seizure 6-like protein 2 OS=Homo sapiens OX=9606 GN=SEZ6L2 PE=1 SV=2	4402.43	5651.51
INO80 complex subunit D OS=Homo sapiens OX=9606 GN=INO80D PE=1 SV=2	5.25E+04	6.34E+04
Ras-like protein family member 11A OS=Homo sapiens OX=9606 GN=RASL11A PE=2 SV=1	---	---
Dual specificity protein phosphatase 8 OS=Homo sapiens OX=9606 GN=DUSP8 PE=1 SV=2	---	---
Interleukin-31 receptor subunit alpha OS=Homo sapiens OX=9606 GN=IL31RA PE=1 SV=1	---	---
EMILIN-2 OS=Homo sapiens OX=9606 GN=EMILIN2 PE=1 SV=3	---	---
Cardiomyopathy-associated protein 5 OS=Homo sapiens OX=9606 GN=CMYA5 PE=1 SV=3	1.08E+06	8.09E+05
Propionyl-CoA carboxylase beta chain, mitochondrial OS=Homo sapiens OX=9606 GN=PCCB PE=1 SV=3	2.57E+05	2.35E+05
Bromodomain adjacent to zinc finger domain protein 2A OS=Homo sapiens OX=9606 GN=BAZ2A PE=1 SV=4	---	---
CWF19-like protein 2 OS=Homo sapiens OX=9606 GN=CWF19L2 PE=1 SV=4	---	---
Plectin OS=Homo sapiens OX=9606 GN=PLEC PE=1 SV=3	---	---
Uncharacterized protein C19orf57 OS=Homo sapiens OX=9606 GN=C19orf57 PE=1 SV=2	5.88E+05	4.14E+05
Ribosomal protein S6 kinase alpha-3 OS=Homo sapiens OX=9606 GN=RPS6KA3 PE=1 SV=1	3.44E+04	3.40E+04
B-cell receptor CD22 OS=Homo sapiens OX=9606 GN=CD22 PE=1 SV=2	3.04E+05	2.85E+05
Retinol dehydrogenase 14 OS=Homo sapiens OX=9606 GN=RDH14 PE=1 SV=1	1.26E+04	1.25E+04
Neuroserpin OS=Homo sapiens OX=9606 GN=SERPINI1 PE=1 SV=1	4.77E+04	4.39E+04
Transmembrane protein 212 OS=Homo sapiens OX=9606 GN=TMEM212 PE=2 SV=2	3.05E+04	2.77E+04
Receptor-type tyrosine-protein phosphatase U OS=Homo sapiens OX=9606 GN=PTPRU PE=1 SV=2	3.36E+04	2.80E+04
Ubiquitin carboxyl-terminal hydrolase 36 OS=Homo sapiens OX=9606 GN=USP36 PE=1 SV=4	---	---
Endonuclease V OS=Homo sapiens OX=9606 GN=ENDOV PE=1 SV=1	1.12E+04	1.24E+04
Sushi domain-containing protein 5 OS=Homo sapiens OX=9606 GN=SUSD5 PE=1 SV=3	2407.85	3140.15
Ephrin type-A receptor 4 OS=Homo sapiens OX=9606 GN=EPHA4 PE=1 SV=1	2980.02	2361.17

Tetratricopeptide repeat protein 7A OS=Homo sapiens OX=9606 GN=TTC7A PE=1 SV=3	5.27E+04	4.13E+04
Cadherin-18 OS=Homo sapiens OX=9606 GN=CDH18 PE=2 SV=1	---	---
Calcium uptake protein 3, mitochondrial OS=Homo sapiens OX=9606 GN=MICU3 PE=2 SV=1	4.40E+04	5.03E+04
Insulin-like growth factor II OS=Homo sapiens OX=9606 GN=IGF2 PE=1 SV=1	8.44E+04	8.42E+04
Bone sialoprotein 2 OS=Homo sapiens OX=9606 GN=IBSP PE=1 SV=4	6.70E+05	7.31E+05
Immunoglobulin lambda variable 3-10 OS=Homo sapiens OX=9606 GN=IGLV3-10 PE=3 SV=2	4279.97	2600.61
Regulator of nonsense transcripts 3A OS=Homo sapiens OX=9606 GN=UPF3A PE=1 SV=1	4399.4	4480.4
Beta-klotho OS=Homo sapiens OX=9606 GN=KLB PE=1 SV=1	3.08E+05	2.94E+05
Symplekin OS=Homo sapiens OX=9606 GN=SYMPK PE=1 SV=2	2.32E+04	2.16E+04
Neurogenic locus notch homolog protein 3 OS=Homo sapiens OX=9606 GN=NOTCH3 PE=1 SV=2	2.30E+05	1.96E+05
DNA-directed RNA polymerase II subunit RPB1 OS=Homo sapiens OX=9606 GN=POLR2A PE=1 SV=2	1.81E+04	1.94E+04
POTE ankyrin domain family member I OS=Homo sapiens OX=9606 GN=POTEI PE=3 SV=1	---	---
Rho guanine nucleotide exchange factor 17 OS=Homo sapiens OX=9606 GN=ARHGEF17 PE=1 SV=1	---	---
Junction plakoglobin OS=Homo sapiens OX=9606 GN=JUP PE=1 SV=3	1.47E+05	1.21E+05
DNA replication ATP-dependent helicase/nuclease DNA2 OS=Homo sapiens OX=9606 GN=DNA2 PE=1 SV=3	1.12E+04	1.34E+04
Zinc fingers and homeoboxes protein 2 OS=Homo sapiens OX=9606 GN=ZHX2 PE=1 SV=1	7.08E+04	8.19E+04
Band 4.1-like protein 2 OS=Homo sapiens OX=9606 GN=EPB41L2 PE=1 SV=1	3.48E+04	4.23E+04
Membrane-associated phosphatidylinositol transfer protein 2 OS=Homo sapiens OX=9606 GN=PITPNM2 PE=1 SV=1	2.90E+05	2.73E+05
MHC class II regulatory factor RFX1 OS=Homo sapiens OX=9606 GN=RFX1 PE=1 SV=2	---	---
Synaptic vesicle glycoprotein 2A OS=Homo sapiens OX=9606 GN=SV2A PE=1 SV=1	5.34E+04	4.61E+04
Isocitrate dehydrogenase [NAD] subunit gamma, mitochondrial OS=Homo sapiens OX=9606 GN=IDH3G PE=1 SV=1	---	---

Little elongation complex subunit 2 OS=Homo sapiens OX=9606 GN=ICE2 PE=1 SV=2	---	---
Dystrophin OS=Homo sapiens OX=9606 GN=DMD PE=1 SV=3	1.67E+05	1.63E+05
Inositol hexakisphosphate and diphosphoinositol-pentakisphosphate kinase 2 OS=Homo sapiens OX=9606 GN=PPIP5K2 PE=1 SV=3	1.28E+06	8.50E+05
Transmembrane anterior posterior transformation protein 1 homolog OS=Homo sapiens OX=9606 GN=TAPT1 PE=1 SV=1	---	---
Macrophage colony-stimulating factor 1 OS=Homo sapiens OX=9606 GN=CSF1 PE=1 SV=2	8651.66	8855.94
Sorting nexin-14 OS=Homo sapiens OX=9606 GN=SNX14 PE=1 SV=3	2.55E+05	2.62E+05
Serine/threonine-protein kinase LMTK2 OS=Homo sapiens OX=9606 GN=LMTK2 PE=1 SV=2	5.10E+04	5.07E+04
Tetratricopeptide repeat protein 36 OS=Homo sapiens OX=9606 GN=TTC36 PE=1 SV=1	6.30E+05	5.39E+05
Nebulette OS=Homo sapiens OX=9606 GN=NEBL PE=1 SV=1	1484.37	1824.09
Zinc finger protein 43 OS=Homo sapiens OX=9606 GN=ZNF43 PE=2 SV=4	1.45E+05	1.70E+05
Sarcolemmal membrane-associated protein OS=Homo sapiens OX=9606 GN=SLMAP PE=1 SV=1	4.25E+04	3.46E+04
HEAT repeat-containing protein 1 OS=Homo sapiens OX=9606 GN=HEATR1 PE=1 SV=3	3.27E+06	3.13E+06
Aminoacyl tRNA synthase complex-interacting multifunctional protein 1 OS=Homo sapiens OX=9606 GN=AIMP1 PE=1 SV=2	3.24E+06	2.67E+06
VPS10 domain-containing receptor SorCS2 OS=Homo sapiens OX=9606 GN=SORCS2 PE=1 SV=3	2.56E+05	2.38E+05
AT-rich interactive domain-containing protein 3B OS=Homo sapiens OX=9606 GN=ARID3B PE=1 SV=2	1.25E+04	8342.8
DNA endonuclease RBBP8 OS=Homo sapiens OX=9606 GN=RBBP8 PE=1 SV=2	3.95E+05	3.14E+05
Microtubule-associated protein 1A OS=Homo sapiens OX=9606 GN=MAP1A PE=1 SV=6	6.37E+05	6.73E+05
WD repeat-containing protein 87 OS=Homo sapiens OX=9606 GN=WDR87 PE=1 SV=3	2.86E+04	3.64E+04
Zinc finger protein 449 OS=Homo sapiens OX=9606 GN=ZNF449 PE=1 SV=3	5.43E+04	3.70E+04
Bcl-2-like protein 13 OS=Homo sapiens OX=9606 GN=BCL2L13 PE=1 SV=1	3.67E+06	3.21E+06
Protocadherin beta-16 OS=Homo sapiens OX=9606 GN=PCDHB16 PE=1 SV=3	7.80E+04	5.50E+04

Olfactory receptor 1N2 OS=Homo sapiens OX=9606 GN=OR1N2 PE=2 SV=2	2.27E+04	2.56E+04
Transmembrane protein 191B OS=Homo sapiens OX=9606 GN=TMEM191B PE=3 SV=2	476.56	2563.5
CD2-associated protein OS=Homo sapiens OX=9606 GN=CD2AP PE=1 SV=1	4.22E+06	4.94E+06
Serine/threonine-protein phosphatase 2B catalytic subunit beta isoform OS=Homo sapiens OX=9606 GN=PPP3CB PE=1 SV=2	1.46E+05	1.04E+05
Glucokinase regulatory protein OS=Homo sapiens OX=9606 GN=GCKR PE=1 SV=6	3.10E+04	2.16E+04
Transcriptional repressor p66-alpha OS=Homo sapiens OX=9606 GN=GATAD2A PE=1 SV=1	4.92E+05	5.62E+05
Golgin subfamily A member 2 OS=Homo sapiens OX=9606 GN=GOLGA2 PE=1 SV=3	2.35E+05	2.38E+05
Keratin, type II cytoskeletal 74 OS=Homo sapiens OX=9606 GN=KRT74 PE=1 SV=2	3.13E+05	3.66E+05
Fos-related antigen 2 OS=Homo sapiens OX=9606 GN=FOSL2 PE=1 SV=1	3.03E+06	3.03E+06
RING finger protein 113A OS=Homo sapiens OX=9606 GN=RNF113A PE=1 SV=1	---	---
BAH and coiled-coil domain-containing protein 1 OS=Homo sapiens OX=9606 GN=BAHCC1 PE=1 SV=4	3.06E+04	2.99E+04
Neuron navigator 3 OS=Homo sapiens OX=9606 GN=NAV3 PE=1 SV=3	5261.87	1.03E+04
Cadherin-22 OS=Homo sapiens OX=9606 GN=CDH22 PE=2 SV=2	2.93E+05	2.40E+05
Adapter molecule crk OS=Homo sapiens OX=9606 GN=CRK PE=1 SV=2	3.32E+04	4.44E+04
Leucine-rich repeat and IQ domain-containing protein 1 OS=Homo sapiens OX=9606 GN=LRRIQ1 PE=2 SV=3	1.70E+04	1.79E+04
Kv channel-interacting protein 2 OS=Homo sapiens OX=9606 GN=KCNIP2 PE=1 SV=3	2.12E+05	1.81E+05
Uncharacterized protein C9orf163 OS=Homo sapiens OX=9606 GN=C9orf163 PE=2 SV=1	---	---
Putative gametogenetin-binding protein 1 OS=Homo sapiens OX=9606 GN=GGNBP1 PE=5 SV=1	2.38E+04	2.54E+04
Dystonin OS=Homo sapiens OX=9606 GN=DST PE=1 SV=4	---	---
DDB1- and CUL4-associated factor 8-like protein 2 OS=Homo sapiens OX=9606 GN=DCAF8L2 PE=2 SV=2	1.02E+06	9.08E+05
Sodium-dependent noradrenaline transporter OS=Homo sapiens OX=9606 GN=SLC6A2 PE=1 SV=1	1.07E+04	7790.05
Adenylate kinase isoenzyme 6 OS=Homo sapiens OX=9606 GN=AK6 PE=1 SV=1	1.53E+05	1.18E+05

Zinc finger protein 436 OS=Homo sapiens OX=9606 GN=ZNF436 PE=1 SV=2	1.64E+04	1.26E+04
Prorelaxin H2 OS=Homo sapiens OX=9606 GN=RLN2 PE=1 SV=1	1.71E+05	1.10E+05
Cytohesin-2 OS=Homo sapiens OX=9606 GN=CYTH2 PE=1 SV=2	---	---
Oxysterol-binding protein-related protein 10 OS=Homo sapiens OX=9606 GN=OSBPL10 PE=1 SV=2	1.30E+05	1.21E+05
Neutral alpha-glucosidase AB OS=Homo sapiens OX=9606 GN=GANAB PE=1 SV=3	1332.8	851.54
Copine-8 OS=Homo sapiens OX=9606 GN=CPNE8 PE=1 SV=2	2.11E+06	1.36E+06
HMG box transcription factor BBX OS=Homo sapiens OX=9606 GN=BBX PE=1 SV=1	1.90E+04	2.18E+04
Pre-mRNA-splicing factor SYF1 OS=Homo sapiens OX=9606 GN=XAB2 PE=1 SV=2	---	---
T cell receptor beta variable 13 OS=Homo sapiens OX=9606 GN=TRBV13 PE=3 SV=5	6.34E+04	9.54E+04
Endothelin-converting enzyme 2 OS=Homo sapiens OX=9606 GN=ECE2 PE=1 SV=1	2.37E+05	1.95E+05
Microtubule-associated serine/threonine-protein kinase 3 OS=Homo sapiens OX=9606 GN=MAST3 PE=1 SV=2	9.67E+04	8.73E+04
Lymphocyte cytosolic protein 2 OS=Homo sapiens OX=9606 GN=LCP2 PE=1 SV=1	1.36E+04	2.08E+04
Deleted in autism protein 1 OS=Homo sapiens OX=9606 GN=C3orf58 PE=1 SV=1	---	---
Potassium/sodium hyperpolarization-activated cyclic nucleotide-gated channel 2 OS=Homo sapiens OX=9606 GN=HCN2 PE=1 SV=3	1.36E+06	9.08E+05
Serine/threonine-protein kinase 31 OS=Homo sapiens OX=9606 GN=STK31 PE=2 SV=2	3.25E+04	3.30E+04
TSC22 domain family protein 2 OS=Homo sapiens OX=9606 GN=TSC22D2 PE=1 SV=3	1.15E+07	9.95E+06
T-box brain protein 1 OS=Homo sapiens OX=9606 GN=TBR1 PE=1 SV=1	---	---
Pleckstrin homology domain-containing family A member 2 OS=Homo sapiens OX=9606 GN=PLEKHA2 PE=1 SV=2	1.36E+04	1.52E+04
Protein phosphatase 1 regulatory subunit 42 OS=Homo sapiens OX=9606 GN=PPP1R42 PE=2 SV=3	---	---
Acetyl-CoA acetyltransferase, cytosolic OS=Homo sapiens OX=9606 GN=ACAT2 PE=1 SV=2	2.57E+05	3.19E+05
Meiotic recombination protein SPO11 OS=Homo sapiens OX=9606 GN=SPO11 PE=2 SV=1	4741.9	6190.74

Death-associated protein kinase 3 OS=Homo sapiens OX=9606 GN=DAPK3 PE=1 SV=1	4425.27	2685.64
Ataxin-3 OS=Homo sapiens OX=9606 GN=ATXN3 PE=1 SV=5	8.56E+04	8.88E+04
Fibrillin-1 OS=Homo sapiens OX=9606 GN=FBN1 PE=1 SV=3	1.15E+05	1.15E+05
Intersectin-1 OS=Homo sapiens OX=9606 GN=ITSN1 PE=1 SV=3	2.06E+05	1.90E+05
TRMT1-like protein OS=Homo sapiens OX=9606 GN=TRMT1L PE=1 SV=2	---	---
Protein Jade-1 OS=Homo sapiens OX=9606 GN=JADE1 PE=1 SV=1	5.08E+04	4.61E+04
Protein cordon-bleu OS=Homo sapiens OX=9606 GN=COBL PE=1 SV=2	8.89E+04	1.42E+05
Malignant fibrous histiocytoma-amplified sequence 1 OS=Homo sapiens OX=9606 GN=MFHAS1 PE=1 SV=2	2.76E+04	2.55E+04
ATP-dependent RNA helicase DHX8 OS=Homo sapiens OX=9606 GN=DHX8 PE=1 SV=1	8697.88	1.15E+04
Failed axon connections homolog OS=Homo sapiens OX=9606 GN=FAXC PE=2 SV=2	---	---
Triosephosphate isomerase OS=Homo sapiens OX=9606 GN=TPI1 PE=1 SV=3	4.25E+05	2.17E+05
Iduronate 2-sulfatase OS=Homo sapiens OX=9606 GN=IDS PE=1 SV=1	2.55E+04	2.63E+04
Leucine-rich repeat serine/threonine-protein kinase 2 OS=Homo sapiens OX=9606 GN=LRRK2 PE=1 SV=2	1.75E+05	1.54E+05
PR domain zinc finger protein 8 OS=Homo sapiens OX=9606 GN=PRDM8 PE=1 SV=3	1.20E+06	9.39E+05
Neuroligin-1 OS=Homo sapiens OX=9606 GN=NLGN1 PE=1 SV=2	1.09E+06	9.52E+05
Growth hormone-regulated TBC protein 1 OS=Homo sapiens OX=9606 GN=GRTP1 PE=1 SV=4	7.02E+05	6.71E+05
LINE-1 retrotransposable element ORF2 protein OS=Homo sapiens OX=9606 PE=1 SV=1	5.66E+04	5.76E+04
Poly [ADP-ribose] polymerase 4 OS=Homo sapiens OX=9606 GN=PARP4 PE=1 SV=3	---	---
Signal transducer and activator of transcription 2 OS=Homo sapiens OX=9606 GN=STAT2 PE=1 SV=1	1.06E+04	9755.27
Integrin alpha-L OS=Homo sapiens OX=9606 GN=ITGAL PE=1 SV=3	2.13E+04	1.82E+04
Collagen alpha-1(XXIII) chain OS=Homo sapiens OX=9606 GN=COL23A1 PE=1 SV=1	2.75E+06	1.98E+06
Gliomedin OS=Homo sapiens OX=9606 GN=GLDN PE=1 SV=1	2.31E+05	2.30E+05

Protein Niban OS=Homo sapiens OX=9606 GN=FAM129A PE=1 SV=1	1.80E+05	1.58E+05
Guanine nucleotide exchange factor for Rab-3A OS=Homo sapiens OX=9606 GN=RAB3IL1 PE=1 SV=1	1.48E+05	1.10E+05
Very long-chain acyl-CoA synthetase OS=Homo sapiens OX=9606 GN=SLC27A2 PE=1 SV=2	---	---
Thyroid hormone receptor beta OS=Homo sapiens OX=9606 GN=THRB PE=1 SV=2	1.35E+06	1.33E+06
Dynein heavy chain 6, axonemal OS=Homo sapiens OX=9606 GN=DNAH6 PE=2 SV=3	4726.32	5400.91
Protein phosphatase 1E OS=Homo sapiens OX=9606 GN=PPM1E PE=1 SV=3	2.73E+07	2.80E+07
GRB10-interacting GYF protein 2 OS=Homo sapiens OX=9606 GN=GIGYF2 PE=1 SV=1	2.25E+05	2.58E+05
Methyl-CpG-binding protein 2 OS=Homo sapiens OX=9606 GN=MECP2 PE=1 SV=1	1.02E+06	1.23E+06
ATP-dependent 6-phosphofructokinase, muscle type OS=Homo sapiens OX=9606 GN=PFKM PE=1 SV=2	1.27E+05	6.95E+04
Treacle protein OS=Homo sapiens OX=9606 GN=TCOF1 PE=1 SV=3	8.71E+06	6.51E+06
Sacsin OS=Homo sapiens OX=9606 GN=SACS PE=1 SV=2	5.50E+05	6.66E+05
NXPE family member 3 OS=Homo sapiens OX=9606 GN=NXPE3 PE=2 SV=1	1.15E+04	1.92E+04
Sorting nexin-13 OS=Homo sapiens OX=9606 GN=SNX13 PE=1 SV=4	3.55E+04	4.30E+04
Transmembrane protein with metallophosphoesterase domain OS=Homo sapiens OX=9606 GN=TMPPE PE=2 SV=2	954.74	761.55
