# Supplementary material for: Cerebrospinal fluid proteome evaluation in major depressive disorder by mass spectrometry
Source: BMC Psychiatry. 2020 Oct 1;20:481. doi: 10.1186/s12888-020-02874-9 (PMC7528485; doi:10.1186/s12888-020-02874-9)
Supplement: Supplementary file 3 — Additional file 3: Supplementary Table 3. A complete list of all proteins identified by SWATH analysis. [file 12888_2020_2874_MOESM3_ESM.pdf]

UniprotID	Accession	MDD/CONT	Zpval
**IBP2_HUMAN**	P18065	0.77913036	2.220E-16
**A2AP_HUMAN**	P08697	1.282274726	4.441E-16
**PI16_HUMAN**	Q6UXB8	5.663648847	6.661E-16
**RTN4R_HUMAN**	Q9BZR6	1.453599349	1.110E-15
**IC1_HUMAN**	P05155	0.740730404	6.217E-15
**SODC_HUMAN**	P00441	1.100725663	6.839E-14
**B2MG_HUMAN**	P61769	1.243884388	3.164E-13
**ENDD1_HUMAN**	O94919	1.366511171	3.904E-13
**YIPF3_HUMAN**	Q9GZM5	0.638057755	4.090E-13
**CO1A1_HUMAN**	P02452	0.675602924	5.274E-13
**APOA4_HUMAN**	P06727	1.428990674	8.440E-13
**CLUS_HUMAN**	P10909	1.313725012	1.114E-12
**ATRN_HUMAN**	O75882	0.840242052	2.086E-12
**DPP2_HUMAN**	Q9UHL4	1.697838351	3.307E-12
**NCAM2_HUMAN**	O15394	1.241423636	4.132E-12
**CO4B_HUMAN**	P0C0L5	0.521955704	7.015E-12
**APOD_HUMAN**	P05090	0.786508172	5.860E-11
**PLMN_HUMAN**	P00747	1.702902707	6.259E-11
**NCAN_HUMAN**	O14594	0.744441696	2.658E-10
**NRX3A_HUMAN**	Q9Y4C0	0.550915697	2.944E-10
**GLU2B_HUMAN**	P14314	0.195515085	3.570E-10
**CADM2_HUMAN**	Q8N3J6	0.683634315	3.973E-10
**CADH2_HUMAN**	P19022	1.285563942	8.727E-10
**CO9_HUMAN**	P02748	1.557499445	9.958E-10
**NRX2A_HUMAN**	Q9P2S2	0.889480121	1.219E-09
**IGHG4_HUMAN**	P01861	0.498247848	1.302E-09
**ANGT_HUMAN**	P01019	0.784788528	1.866E-09
**CFAH_HUMAN**	P08603	0.793401982	2.204E-09
**CBPE_HUMAN**	P16870	0.721895301	2.916E-09
**CALR_HUMAN**	P27797	0.707754955	5.351E-09
**CHL1_HUMAN**	O00533	0.645741518	7.901E-09
**LSAMP_HUMAN**	Q13449	1.517592435	8.690E-09
**ITIH4_HUMAN**	Q14624	1.438330927	8.983E-09
**KV303_HUMAN**	P01621	1.405342338	1.184E-08
**LAC3_HUMAN**	P0CG06	0.539664965	1.839E-08
**LV107_HUMAN**	P06316	0.713212191	1.898E-08
**KLK6_HUMAN**	Q92876	1.139726824	3.300E-08
**FAM3C_HUMAN**	Q92520	0.7221056	6.729E-08
**ITIH1_HUMAN**	P19827	1.49422601	7.878E-08
**OMGP_HUMAN**	P23515	1.900149401	1.442E-07
**AACT_HUMAN**	P01011	1.107215607	2.000E-07
**RNAS1_HUMAN**	P07998	0.696963975	3.458E-07
**CYC_HUMAN**	P99999	1.440492531	4.117E-07
**SCG3_HUMAN**	Q8WXD2	0.786957962	4.642E-07
**ANT3_HUMAN**	P01008	0.667210308	7.541E-07
**CFAI_HUMAN**	P05156	1.499421917	8.643E-07

**OPCM_HUMAN**	Q14982	1.424047652	2.294E-06
**NPDC1_HUMAN**	Q9NQX5	1.187508378	2.554E-06
**HV103_HUMAN**	P23083	0.678681176	8.579E-06
**AMBP_HUMAN**	P02760	1.105418121	1.005E-05
**IGHG1_HUMAN**	P01857	0.759327488	1.362E-05
**PGBM_HUMAN**	P98160	0.470977573	1.643E-05
**WFKN2_HUMAN**	Q8TEU8	1.241436514	1.946E-05
**CA2D1_HUMAN**	P54289	0.716799429	3.959E-05
**LCAT_HUMAN**	P04180	0.654232678	4.629E-05
**CFAB_HUMAN**	P00751	1.341532133	5.987E-05
**APOE_HUMAN**	P02649	0.793386513	6.492E-05
**CATD_HUMAN**	P07339	0.299549126	9.144E-05
**BAI2_HUMAN**	O60241	1.317816754	1.424E-04
**FA12_HUMAN**	P00748	1.497586923	1.509E-04
**ANFC_HUMAN**	P23582	1.111617015	2.268E-04
**MUC18_HUMAN**	P43121	0.663660811	2.767E-04
**SPRL1_HUMAN**	Q14515	0.702478365	3.208E-04
**CD166_HUMAN**	Q13740	0.632606489	3.394E-04
**NTRI_HUMAN**	Q9P121	1.123936554	5.887E-04
**LTBP4_HUMAN**	Q8N2S1	0.56576499	6.542E-04
**FBLN1_HUMAN**	P23142	0.82194967	9.706E-04
**SE6L1_HUMAN**	Q9BYH1	0.411278425	9.807E-04
**TIMP2_HUMAN**	P16035	1.132244203	1.001E-03
**NTRK2_HUMAN**	Q16620	1.406289945	1.222E-03
**FIBA_HUMAN**	P02671	1.228118417	1.586E-03
**PTPR2_HUMAN**	Q92932	1.091983713	1.658E-03
**HV209_HUMAN**	P06331	0.876124772	1.675E-03
**ALDOC_HUMAN**	P09972	0.488842007	1.679E-03
**IGHM_HUMAN**	P01871	1.872226758	1.795E-03
**CO8G_HUMAN**	P07360	0.460609712	2.522E-03
**PGS2_HUMAN**	P07585	1.760911114	2.846E-03
**CD59_HUMAN**	P13987	1.083541492	3.133E-03
**IGKC_HUMAN**	P01834	1.077738068	3.635E-03
**ALDOA_HUMAN**	P04075	1.07926388	4.254E-03
**7B2_HUMAN**	P05408	0.621439757	4.520E-03
**DKK3_HUMAN**	Q9UBP4	0.895707159	5.061E-03
**A2GL_HUMAN**	P02750	3.131352545	5.679E-03
**A1AG2_HUMAN**	P19652	1.193410375	5.815E-03
**HEP2_HUMAN**	P05546	0.996392872	6.829E-03
**KLKB1_HUMAN**	P03952	1.394976617	6.939E-03
**C1S_HUMAN**	P09871	0.48079864	7.094E-03
**CSPG5_HUMAN**	O95196	0.711575915	8.343E-03
**RELN_HUMAN**	P78509	0.703614598	8.718E-03
**ITIH2_HUMAN**	P19823	1.329780981	8.724E-03
**BTD_HUMAN**	P43251	1.303385133	1.088E-02
**IGHG2_HUMAN**	P01859	0.868583933	1.210E-02
**K1C9_HUMAN**	P35527	1.209199522	1.217E-02

**CMGA_HUMAN**	P10645	0.832170135	1.413E-02
**B3GN1_HUMAN**	O43505	0.898822061	1.427E-02
**SCG1_HUMAN**	P05060	1.051750188	1.533E-02
**CP089_HUMAN**	Q6UX73	1.610403827	1.660E-02
**AATC_HUMAN**	P17174	0.476918642	1.831E-02
**NPTX1_HUMAN**	Q15818	0.324519447	2.303E-02
**PLTP_HUMAN**	P55058	0.344153562	2.557E-02
**ROBO1_HUMAN**	Q9Y6N7	0.536254015	2.566E-02
**KV205_HUMAN**	P06309	1.033509435	2.950E-02
**CYTC_HUMAN**	P01034	0.863164852	3.100E-02
**CNTN1_HUMAN**	Q12860	0.714226497	3.251E-02
**TICN2_HUMAN**	Q92563	0.974132934	3.256E-02
**NPC2_HUMAN**	P61916	1.112824057	3.396E-02
**MEGF8_HUMAN**	Q7Z7M0	1.318976289	3.437E-02
**LV302_HUMAN**	P80748	0.695560349	3.488E-02
**LV301_HUMAN**	P01714	8.427654886	3.519E-02
**TICN1_HUMAN**	Q08629	1.312250801	3.629E-02
**C1QB_HUMAN**	P02746	0.806250111	3.725E-02
**C1QC_HUMAN**	P02747	0.72520993	3.803E-02
**KV404_HUMAN**	P06314	0.741522802	3.812E-02
**LDHB_HUMAN**	P07195	0.472074202	4.138E-02
**OSTP_HUMAN**	P10451	1.00702726	4.275E-02
**NFASC_HUMAN**	O94856	0.754350654	4.303E-02
**CADM1_HUMAN**	Q9BY67	1.261293934	4.670E-02
**APOA2_HUMAN**	P02652	1.181318623	4.997E-02
**PENK_HUMAN**	P01210	1.138095125	5.024E-02
**TIMP1_HUMAN**	P01033	0.703190189	5.213E-02
**SULF2_HUMAN**	Q8IWU5	0.559836861	5.385E-02
**K22E_HUMAN**	P35908	1.329792084	5.985E-02
**HEXB_HUMAN**	P07686	0.355440998	6.050E-02
**CATB_HUMAN**	P07858	0.320423217	6.177E-02
**NEUS_HUMAN**	Q99574	0.574802142	6.426E-02
**PTPRD_HUMAN**	P23468	1.308637761	6.707E-02
**CD14_HUMAN**	P08571	0.875711193	6.779E-02
**MIME_HUMAN**	P20774	1.036534619	7.122E-02
**P3IP1_HUMAN**	Q96FE7	1.592885147	7.167E-02
**CO7_HUMAN**	P10643	0.831602159	7.790E-02
**IBP4_HUMAN**	P22692	1.582017432	7.903E-02
**HV318_HUMAN**	P01779	1.737578368	8.187E-02
**THBG_HUMAN**	P05543	0.498355435	8.292E-02
**CART_HUMAN**	Q16568	0.469224042	8.372E-02
**CO6_HUMAN**	P13671	1.82902964	8.414E-02
**CO4A_HUMAN**	P0C0L4	1.348285209	8.667E-02
**CSF1_HUMAN**	P09603	0.56027802	9.737E-02
**C99L2_HUMAN**	Q8TCZ2	1.361153023	1.043E-01
**SE6L2_HUMAN**	Q6UXD5	1.66981564	1.078E-01
**GOLM1_HUMAN**	Q8NBJ4	0.82361895	1.147E-01

**PEBP4_HUMAN**	Q96S96	0.491404621	1.191E-01
**VAS1_HUMAN**	Q15904	1.118378228	1.216E-01
**KV307_HUMAN**	P04206	0.975055767	1.220E-01
**ALBU_HUMAN**	P02768	1.040211038	1.233E-01
**CALY_HUMAN**	Q9NYX4	1.438293044	1.299E-01
**SHPS1_HUMAN**	P78324	1.15429476	1.309E-01
**BGH3_HUMAN**	Q15582	0.698936158	1.310E-01
**PON1_HUMAN**	P27169	1.285185577	1.329E-01
**SCRG1_HUMAN**	O75711	2.623281099	1.370E-01
**KV119_HUMAN**	P01611	0.836444655	1.407E-01
**TETN_HUMAN**	P05452	0.892897215	1.482E-01
**CO6A1_HUMAN**	P12109	1.00766193	1.598E-01
**UFO_HUMAN**	P30530	1.388920508	1.754E-01
**KV309_HUMAN**	P04433	1.418579759	1.779E-01
**IGF2_HUMAN**	P01344	0.829879058	1.795E-01
**AFAM_HUMAN**	P43652	1.233994383	1.800E-01
**NEO1_HUMAN**	Q92859	0.827783474	1.996E-01
**LV106_HUMAN**	P04208	0.652839525	1.999E-01
**SEPP1_HUMAN**	P49908	0.288588747	2.037E-01
**LMAN2_HUMAN**	Q12907	0.981100416	2.134E-01
**A2MG_HUMAN**	P01023	0.897271993	2.281E-01
**PRIO_HUMAN**	P04156	0.821634623	2.303E-01
**COIA1_HUMAN**	P39060	0.872100531	2.321E-01
**NPTXR_HUMAN**	O95502	1.049778388	2.355E-01
**SCG2_HUMAN**	P13521	1.023500123	2.502E-01
**IDS_HUMAN**	P22304	1.336339309	2.518E-01
**THIO_HUMAN**	P10599	1.155560117	2.573E-01
**CATL1_HUMAN**	P07711	1.14077163	2.713E-01
**NRCAM_HUMAN**	Q92823	0.90852085	2.798E-01
**NEGR1_HUMAN**	Q7Z3B1	1.033808256	2.803E-01
**R4RL2_HUMAN**	Q86UN3	0.641633815	2.813E-01
**PTPRZ_HUMAN**	P23471	1.034940176	2.833E-01
**MOG_HUMAN**	Q16653	1.039942641	2.947E-01
**IBP6_HUMAN**	P24592	0.781395974	2.969E-01
**NRX1A_HUMAN**	Q9ULB1	0.820078191	3.016E-01
**TPIS_HUMAN**	P60174	1.024107349	3.188E-01
**PTGDS_HUMAN**	P41222	0.945987208	3.403E-01
**G3P_HUMAN**	P04406	0.763229728	3.469E-01
**PGRP2_HUMAN**	Q96PD5	1.201419283	3.495E-01
**SBP1_HUMAN**	Q13228	0.723891411	3.531E-01
**KPYM_HUMAN**	P14618	0.987220297	3.556E-01
**CNTN2_HUMAN**	Q02246	0.760891935	3.599E-01
**CO8A_HUMAN**	P07357	1.07391404	3.610E-01
**CO1A2_HUMAN**	P08123	1.03134301	3.644E-01
**CO8B_HUMAN**	P07358	1.319673733	3.650E-01
**PTPRN_HUMAN**	Q16849	0.450627741	3.996E-01
**CBPB2_HUMAN**	Q96IY4	1.248815503	4.056E-01

**EPHA4_HUMAN**	P54764	0.893124771	4.133E-01
**SEZ6_HUMAN**	Q53EL9	0.498473339	4.172E-01
**NBL1_HUMAN**	P41271	1.012694306	4.214E-01
**FBLN5_HUMAN**	Q9UBX5	1.316182916	4.233E-01
**CSF1R_HUMAN**	P07333	0.746185794	4.251E-01
**FHR2_HUMAN**	P36980	1.163217251	4.379E-01
**XYLT1_HUMAN**	Q86Y38	1.08793442	4.459E-01
**CADM3_HUMAN**	Q8N126	1.431628052	4.642E-01
**PTPRS_HUMAN**	Q13332	1.081962431	4.769E-01
**CAD13_HUMAN**	P55290	0.992432886	4.842E-01
**HV305_HUMAN**	P01766	1.148998326	5.012E-01
**MA2A1_HUMAN**	Q16706	0.795714686	5.017E-01
**HRG_HUMAN**	P04196	0.676294538	5.195E-01
**OLFL3_HUMAN**	Q9NRN5	0.717646091	5.332E-01
**PMGT1_HUMAN**	Q8WZA1	0.973978095	5.395E-01
**SORC3_HUMAN**	Q9UPU3	0.915599962	5.432E-01
**RNAS4_HUMAN**	P34096	1.050749108	5.699E-01
**LPHN1_HUMAN**	O94910	1.064070553	5.791E-01
**CBPQ_HUMAN**	Q9Y646	1.508745327	5.942E-01
**CSTN3_HUMAN**	Q9BQT9	0.461937094	6.039E-01
**ITIH5_HUMAN**	Q86UX2	0.981840279	6.107E-01
**LRC4B_HUMAN**	Q9NT99	0.872851202	6.190E-01
**PPIB_HUMAN**	P23284	0.908035892	6.321E-01
**CADM4_HUMAN**	Q8NFZ8	0.835054177	6.343E-01
**HV320_HUMAN**	P01781	0.895398719	6.480E-01
**KV203_HUMAN**	P01616	1.003100749	6.499E-01
**SEM7A_HUMAN**	O75326	0.88531357	6.553E-01
**ALS_HUMAN**	P35858	0.951424324	6.627E-01
**MMP2_HUMAN**	P08253	0.868440518	6.665E-01
**DAG1_HUMAN**	Q14118	1.024270093	6.839E-01
**CHLE_HUMAN**	P06276	1.186278589	6.920E-01
**A4_HUMAN**	P05067	0.914684249	7.166E-01
**CO5_HUMAN**	P01031	1.281645585	7.220E-01
**FSTL1_HUMAN**	Q12841	1.418837322	7.249E-01
**GRIA4_HUMAN**	P48058	1.901544724	7.266E-01
**CBLN4_HUMAN**	Q9NTU7	0.965033786	7.299E-01
**SAMP_HUMAN**	P02743	0.232935969	7.316E-01
**ITM2B_HUMAN**	Q9Y287	0.79543546	7.472E-01
**ECM1_HUMAN**	Q16610	0.963741168	7.531E-01
**FBLN3_HUMAN**	Q12805	0.957246858	7.544E-01
**CFAD_HUMAN**	P00746	0.56550728	7.684E-01
**RET4_HUMAN**	P02753	0.93767529	7.773E-01
**SPRC_HUMAN**	P09486	0.787497276	7.896E-01
**AGRIN_HUMAN**	O00468	1.08824984	8.053E-01
**IPSP_HUMAN**	P05154	1.169422806	8.055E-01
**NRN1_HUMAN**	Q9NPD7	0.858703623	8.260E-01
**CATZ_HUMAN**	Q9UBR2	0.921120164	8.562E-01

**CGRE1_HUMAN**	Q99674	0.949140411	8.941E-01
**APOC1_HUMAN**	P02654	0.928763191	8.973E-01
**AMD_HUMAN**	P19021	0.704008187	9.063E-01
**LY6H_HUMAN**	O94772	0.958867092	9.163E-01
**LUM_HUMAN**	P51884	0.938238326	9.309E-01
**NCAM1_HUMAN**	P13591	0.954915577	9.380E-01
**PTPRG_HUMAN**	P23470	0.970821994	9.664E-01
**SPRN_HUMAN**	Q5BIV9	0.893810357	9.873E-01
**ASPG_HUMAN**	P20933	0.290059647	9.970E-01
**CO3_HUMAN**	P01024	1.439554747	0.000E+00
**TRFE_HUMAN**	P02787	1.274537184	0.000E+00
**FINC_HUMAN**	P02751	0.752533514	0.000E+00
**A1AT_HUMAN**	P01009	1.306084446	0.000E+00
**CERU_HUMAN**	P00450	1.466292885	0.000E+00
**ENPP2_HUMAN**	Q13822	0.414403538	0.000E+00
**HEMO_HUMAN**	P02790	0.722097036	0.000E+00
**GELS_HUMAN**	P06396	0.558110182	0.000E+00
**PEDF_HUMAN**	P36955	0.568502007	0.000E+00
**HPT_HUMAN**	P00738	8.984741811	0.000E+00
**THRB_HUMAN**	P00734	1.395573844	0.000E+00
**APOA1_HUMAN**	P02647	2.626789219	0.000E+00
**VTDB_HUMAN**	P02774	1.333815489	0.000E+00
**CNDP1_HUMAN**	Q96KN2	0.694762481	0.000E+00
**HBB_HUMAN**	P68871	0.416786026	0.000E+00
**CSTN1_HUMAN**	O94985	0.574221674	0.000E+00
**TTHY_HUMAN**	P02766	0.543518524	0.000E+00
**NELL2_HUMAN**	Q99435	0.701368394	0.000E+00
**APLP1_HUMAN**	P51693	0.582482108	0.000E+00
**A1BG_HUMAN**	P04217	1.989504112	0.000E+00
**K2C1_HUMAN**	P04264	1.621013696	0.000E+00
**VGF_HUMAN**	O15240	1.98707593	0.000E+00
**IGHA1_HUMAN**	P01876	1.469867965	0.000E+00
**KNG1_HUMAN**	P01042	1.600893014	0.000E+00
**C1R_HUMAN**	P00736	0.631168855	0.000E+00
**FIBB_HUMAN**	P02675	1.410483279	0.000E+00
**CH3L1_HUMAN**	P36222	0.864632244	0.000E+00
**ZA2G_HUMAN**	P25311	1.286548115	0.000E+00
**LG3BP_HUMAN**	Q08380	0.54067222	0.000E+00
**APOH_HUMAN**	P02749	1.472489578	0.000E+00
**CRAC1_HUMAN**	Q9NQ79	0.693656633	0.000E+00
**FIBG_HUMAN**	P02679	1.300809414	0.000E+00
**A1AG1_HUMAN**	P02763	2.303402163	0.000E+00
**K1C10_HUMAN**	P13645	2.261645892	0.000E+00
**PCOC1_HUMAN**	Q15113	0.59028755	0.000E+00
**IBP7_HUMAN**	Q16270	0.651503643	0.000E+00
**FCGBP_HUMAN**	Q9Y6R7	0.303643277	0.000E+00
**SODE_HUMAN**	P08294	0.695918788	0.000E+00

**HBA_HUMAN**	P69905	0.381472851	0.000E+00
**PCSK1_HUMAN**	Q9UHG2	1.181844967	0.000E+00
**GPX3_HUMAN**	P22352	0.565364137	0.000E+00
**VTNC_HUMAN**	P04004	1.236690625	0.000E+00
**SAP_HUMAN**	P07602	1.135015211	0.000E+00
**PGCB_HUMAN**	Q96GW7	0.61124022	0.000E+00
**CBG_HUMAN**	P08185	0.473416424	0.000E+00
**FETUA_HUMAN**	P02765	1.477342516	0.000E+00
**PEBP1_HUMAN**	P30086	1.800533066	0.000E+00
**THY1_HUMAN**	P04216	1.616401641	0.000E+00
**CUTA_HUMAN**	O60888	0.340204323	0.000E+00
**APOC3_HUMAN**	P02656	3.688382511	0.000E+00
**KV116_HUMAN**	P01608	2.299559627	0.000E+00
**IGHG3_HUMAN**	P01860	0.434379804	0.000E+00
**IGSF8_HUMAN**	Q969P0	1.630150469	0.000E+00
**KAIN_HUMAN**	P29622	3.338962897	0.000E+00
**CAH1_HUMAN**	P00915	0.192968153	0.000E+00
**SAP3_HUMAN**	P17900	1.393274595	0.000E+00
**IGHA2_HUMAN**	P01877	1.667262687	0.000E+00
**CD44_HUMAN**	P16070	4.710020235	0.000E+00
**PRDX2_HUMAN**	P32119	0.259150453	0.000E+00
**HBG2_HUMAN**	P69892	0.027730272	0.000E+00
**HV311_HUMAN**	P01772	1.738355474	0.000E+00
**LYVE1_HUMAN**	Q9Y5Y7	0.738932874	0.000E+00
**LV105_HUMAN**	P01703	1.169796994	0.000E+00
**IGJ_HUMAN**	P01591	2.073522164	0
