# Supplementary material for: Cerebrospinal fluid proteome evaluation in major depressive disorder by mass spectrometry
Source: BMC Psychiatry. 2020 Oct 1;20:481. doi: 10.1186/s12888-020-02874-9 (PMC7528485; doi:10.1186/s12888-020-02874-9)
Supplement: Supplementary file 4 — Additional file 4: Supplementary Figure 1. Complete acute phase response signaling. Red nodes are upregulated and green nodes are down regulated. [file 12888_2020_2874_MOESM4_ESM.pdf]

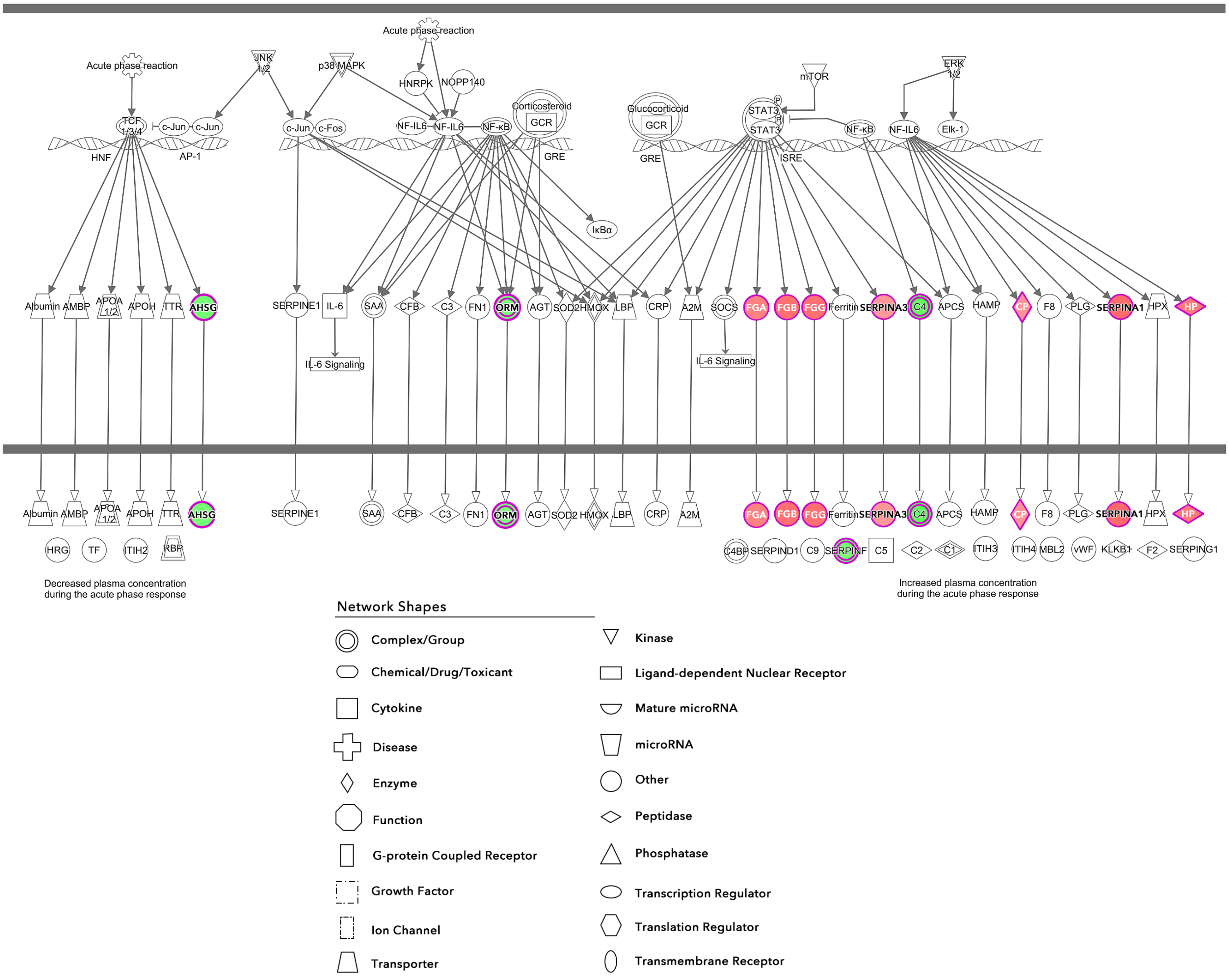
Acute phase reaction
Acute phase reaction
JNK 1/2
p38 MAPK
HNRPK
NOPP140
mTOR
ERK 1/2
TCF 1/3/4
c-Jun
c-Jun
c-Jun
c-Fos
NF-IL6
NF-IL6
NF-κB
Corticosteroid
GCR
Glucocorticoid
GCR
STAT3
STAT3
NF-κB
NF-IL6
Elk-1
HNF
AP-1
GRE
GRE
ISRE
IκBα
Albumin
AMBP
APOA 1/2
APOH
TTR
AHSG
SERPINE1
IL-6
SAA
CFB
C3
FN1
ORM
AGT
SOD2
HMOX
LBP
CRP
A2M
SOCS
FGA
FGB
FGG
Ferritin
SERPINA3
C4
APCS
HAMP
CP
F8
PLG
SERPINA1
HPX
HP
IL-6 Signaling
IL-6 Signaling
HRG
TF
ITIH2
RBP
C4BP
SERPIND1
C9
SERPINF
C5
C2
C1
ITIH3
ITIH4
MBL2
vWF
KLKB1
F2
SERPING1
Decreased plasma concentration during the acute phase response
Increased plasma concentration during the acute phase response
Network Shapes
Complex/Group
Chemical/Drug/Toxicant
Cytokine
Disease
Enzyme
Function
G-protein Coupled Receptor
Growth Factor
Ion Channel
Transporter
Kinase
Ligand-dependent Nuclear Receptor
Mature microRNA
microRNA
Other
Peptidase
Phosphatase
Transcription Regulator
Translation Regulator
Transmembrane Receptor
